# Unveiling the geroprotective potential of *Monarda didyma* L.: insights from in vitro studies and a randomized clinical trial on slowing biological aging and improving quality of life

**DOI:** 10.1007/s11357-025-01580-2

**Published:** 2025-03-10

**Authors:** Manuela Campisi, Luana Cannella, Omar Paccagnella, Alessandra R. Brazzale, Alberto Agnolin, Torsten Grothe, Julia Baumann, Sofia Pavanello

**Affiliations:** 1 Department of Cardiac, Thoracic, and Vascular Sciences and Public Health, University of Padua, Padua, Italy; 2 Department of Statistical Sciences, University of Padua, Padua, Italy; 3 Mibelle Group Biochemistry, Bolimattstrasse 1, Buchs, 5033 Switzerland; 4 Occupational Medicine Unit, University Hospital of Padua, Padua, Italy; 5 Centre of Studies and Activities for Space, CISAS “G. Colombo” of University of Padua, Padua, Italy

**Keywords:** Aging people, Healthy aging, Biological aging, Botanical extracts, DNA methylation age, Leukocyte telomere length, Quality of life

## Abstract

Aging is driven by fundamental mechanisms like oxidative stress, telomere shortening and changes in DNA methylation, which together prepare the ground for age-related diseases. Botanical extracts, rich in bioactive phytoconstituents, represent a promising resource for developing therapies that target these mechanisms to promote healthy aging. This study explores the geroprotective potential of *Monarda didyma* L. extract. In vitro analyses revealed the extract’s strong antioxidant activity, ability to reduce telomere shortening, and capacity to protect against DNA damage, thereby decreasing cellular senescence and improving endothelial function. The randomized, double-blind clinical trial demonstrated that daily oral supplementation with the extract significantly improved leukocyte telomere length (LTL) and stabilized DNA methylation age (DNAmAge) in the intervention group, while the placebo group experienced accelerated epigenetic aging and hypermethylation of critical age-related genes (ELOVL2 and FHL2). The intervention group also reported enhanced quality of life, particularly in the physical domain, along with improved movement and quality sleep indices detected by questionnaire and wearable sensors. These compelling findings position *Monarda didyma* L. extract as a powerful candidate for future geroprotective therapies, with the potential to significantly impact healthy aging.

**Supplementary Information:**

The online version contains supplementary material available at 10.1007/s11357-025-01580-2.

## Introduction

The inexorable progression of population aging poses a significant global challenge, exacerbating social and healthcare burdens worldwide. As the leading risk factor for chronic diseases, aging contributes heavily to global morbidity, mortality, and healthcare costs [1, 2]. The incidence of major chronic diseases—such as ischemic heart disease, stroke, diabetes, liver and kidney diseases, neurodegenerative disorders, and various cancers (including lung and colorectal)—increases with age [3], though the onset and severity of these age-related conditions vary greatly among individuals [4]. Although aging is inevitable, it does not affect everyone in the same way. The aging process, shaped by a unique combination of genetics and environmental factors, can be delayed and compressed through informed lifestyle choices, allowing individuals to live longer at their fullest potential [5, 6]. Elevated oxidative stress and dysregulated inflammation accelerate biological aging, emphasizing the urgent need for safe and effective geroprotective interventions [7, 8].

Recent advancements in aging research have shifted the focus from merely extending lifespan to enhancing healthspan—the period of life spent in good health [9]. Addressing aging as a comprehensive condition, rather than targeting individual age-related diseases, may offer a more impactful approach to mitigating age-related disorders and improving the quality of life for older adults [10, 11]. Central to this strategy is the understanding that age-related cellular damage, such as telomere shortening [12] and aberrant gene methylation, plays a pivotal role in organismal aging [13]. Proteomic age clocks offer innovative diagnostic tools by predicting aging mechanisms through protein analysis [14]. Complementing this, our focus on epigenetics and telomere length provides a dynamic framework for therapeutic interventions, as these mechanisms may be more reversible and actionable for healthspan improvement. DNA methylation and telomere length offer promising and adaptable targets for interventions aimed at slowing or even reversing the aging process. Emerging evidence suggests that interventions aimed at selectively increasing telomere length [15] and modulating gene methylation patterns [16] may not only extend lifespan, but also improve healthspan in aged organisms. Our previous studies demonstrate that intensive relaxation training over 60 days can reverse biological aging markers, improve clinical blood parameters, and enhance endothelial function in both post-myocardial infarction patients and healthy individuals [17]. Additionally, visual art-mediated cognitive activation therapy has been shown to mitigate cellular aging and improve cognitive function in patients with neurocognitive disorders [18].

Botanical extracts have a rich history in traditional, folk, and indigenous medicine for the treating of age-related ailments [19] (https://www.who.int/news-room/feature-stories/detail/traditional-medicine-has-a-long-history-of-contributing-to-conventional-medicine-and-continues-to-hold-promise). Among these, flavonoids—a diverse group of naturally occurring phenolic compounds found in fruits, vegetables, tea, wine, and traditional Chinese medicine [20]—are renowned for their wide-ranging biological activities. These activities include anti-inflammatory [21], vasodilatory [22], anticoagulant [23], cardioprotective [21], anti-diabetic [24, 25], chemoprotective, neuroprotective, anti-obesity [26], and geroprotective effects, coupled with potent antioxidant properties [27].

Didymin (DID), a natural flavonoid glycoside abundant in citrus fruits and in the scarlet beebalm (*Monarda didyma* L.), a mint family herb (Lamiaceae), has garnered significant attention for its multifaceted health benefits [28]. The anti-inflammatory and antioxidant properties of DID [28] make it an ideal candidate for exploring geroprotective effects, particularly those related to early molecular markers of aging such as telomere attrition (indicative of mitotic aging) and alterations in DNA methylation (reflective of non-mitotic aging) [2]. Additionally, DID has shown promise in preventing cellular apoptosis [29] and protecting against mitochondrial dysfunction [30, 31]. Recent studies demonstrated the efficacy of DID in mitigating cadmium-induced renal injury by regulating the Nrf-2/Keap-1 pathway, apoptosis, oxidative stress, and inflammation [32], reducing levels of pro-inflammatory cytokines such as interleukin-1β (IL-1β), IL-18, and tumor necrosis factor α (TNF-α), [33]. These findings further support its potential role in modulating telomere length and DNA methylation dynamics.

In this study, we investigate the geroprotective potential of a standardized extract of *Monarda didyma* L. through both in vitro studies and a clinical trial. Our in vitro results indicate that *Monarda didyma* L. extract enhances markers of cellular aging, including reduced senescence, slower telomere shortening, decreased DNA damage, protein oxidation, improved endothelial function, and reduced vascular permeability. The clinical trial in an aging population was designed to evaluate the effect of oral supplementation with *Monarda didyma* L. extract on DNA methylation age (DNAmAge) and leukocyte telomere length (LTL) while exploring impacts on quality of life and physical parameters, including activity and sleep. Our findings revealed that daily supplementation with *Monarda didyma* L. extract led to improvements in early markers of aging, with an increase in LTL and a maintenance of DNAmAge, and an improvement in perceived quality of life, particularly in the physical domain. Participants in the intervention group also showed improvements in renal function, maintenance of a better hematological condition, and a reduction in inflammation. Building on the established antioxidant and anti-inflammatory properties of DID, this study suggests a new natural, safe, effective, and sustainable candidate to focus on for future therapeutic development aimed at extending healthspan and slowing biological aging. Our study is strengthened by previous research on *Monarda didyma* L*.*, which demonstrated that its essential oil and main component thymol improve learning and memory impairments in aging models by modulating oxidative stress markers and activating Nrf2 and MAPK pathways [34].

## Methods

### *Monarda didyma* L. extract preparation

An amount of 8-kg dried leaves of *Monarda didyma* L. (Valplantes, Switzerland) was used to prepare an extract powder, using 160-kg de-ionized water as extraction solvent. Extraction was performed for 3 h at 85 °C using a DIG-MAZ extractor (Samtec, Austria). After cool-down to 25 °C, the liquid extract was filtrated using a 4-μm depth filter sheet (BEKO K1, Eaton Industries II GmbH, Switzerland). Analysis by ultra-performance liquid chromatography (UPLC) confirmed that DID is the most abundant flavonoid in the extract, with concentrations approximately 80 to 20 times higher than isosakuranetin, the only other flavonoid detected, which was present in trace amounts (0.05–0.2%). No other flavonoids were identified.

To enhance the bioavailability of DID, the extract was formulated with 50% dextrins (Glucidex19, Roquette, France) as a carrier, employing a vacuum evaporation process. This approach, consistent with previous research [35], demonstrated that the bioavailability of DID is nearly threefold higher when formulated with dextrins compared to its free form.

The extract of *Monarda didyma* L. was provided by Mibelle Group Biochemistry (Buchs, Switzerland), according to the ISO 22000:2018 quality standards.

### In vitro studies on *Monarda didyma* L. extract

#### Analysis of protein oxidation

Primary human dermal fibroblasts, isolated from adult donor skin obtained from abdominoplasty surgery according to the previous protocol [36], were grown in 96-well plates in DMEM supplemented with 10% fetal bovine serum (FBS), without antibiotics. The cells were incubated with and without (control) 0.25 mg/mL of *Monarda didyma* L. solution and the reference compound (0.4 mg/mL N-acetylcysteine—NAC) for 24 h, at 37 °C and 5% CO_2_. Subsequently, cells were stressed by the addition of 100 μM H_2_O_2_ for 30 min and then were fixed to the plate wells using an ethanol/acetic acid solution. Protein carbonylation was analyzed by a fluorescent method using multi-well microplate reader (Varioskan, Thermofischer, USA). Experiments were performed in n = 6 replicates per condition.

#### Analysis of telomere length (TL)

Primary human dermal fibroblasts (obtained from adult donor skin, dermal layer, from abdominoplasty surgery) were cultured at 37 °C and 5% CO_2_ in fibroblast medium (HEPES and bicarbonate based, pH 7.4) supplemented with 2% FBS, 1% Fibroblast Growth Supplement and 1% penicillin/streptomcycin solution (Innoprot, Spain) for 72 h. Fibroblasts were then seeded at 5 × 10^3^ cells/cm^2^ prior to experimental start. Cells were treated with a solution of *Monarda didyma* L. extract (0.043 mg/mL), and untreated (control) for a total of 6 weeks, in the above-described culture media. The media and treatment were renewed every 2–3 days and cells passaged at sub-confluence (70–80%) every 7 days. At each passage, cell growth was monitored by counting the cell numbers in an automated Countess™ cell counter (Invitrogen). Experiments were performed in *n* = 3 replicates per condition.

TL analysis (TAT®) was performed with high-throughput (HT) quantitative fluorescence in situ hybridization (Q-FISH) method with a fluorescent (Alexa-488) peptide nucleic acid (PNA) probe (Panagene, South Korea) that recognizes three telomere repeats. In brief, the cells of the respective passages were seeded to clear bottom black-walled 384-well plates at a density of 15 × 10^3^ cells per well, with *n* = 8 replicates per control sample and *n* = 5 replicates per treatment sample. Two identical independent plates were prepared for each sample set. After fixation with methanol/acetic acid (3/1, vol/vol), the cells were treated with pepsin and nuclei processed for in situ hybridization with the PNA probe. After washing and DAPI incubation, the images of the nuclei and telomeres were captured by a high-content screen system (High Content Screening Opera Phenix System, Perkin Elmer) and the intensity of the fluorescent signal, detected from the probes at the 488 nm wavelength, was translated to TL using the Life Length’s proprietary program. For the microscopy analysis, 15 images were captured per well.

#### Analysis of DNA damage-induced aging

Primary human epidermal keratinocytes, isolated from adult donor skin of abdominoplasty surgery according to the previous protocol [37], were cultured at 37 °C and 5% CO_2_ in DermaCult™ Keratinocyte Expansion Medium (STEMCELL Technologies, Switzerland) supplemented with 1% Pen/Strep (100 units/mL penicillin, 100 μg streptomycin). Prior to the experiments, the cells were seeded in 24-well plates (10^5^ cells/cm^2^) directly and on glass coverslips, incubated for 24 h and then pretreated with 1 mg/mL of *Monarda didyma* L. extract solution for another 24 h and untreated as control. The cells were subjected to genotoxic stress induced with 5-bromodeoxyuridine (BrdU) 100 μM and then further incubated. After 48 h, DNA damage response (DDR) markers, such as γH2A.X and H3K9 methylation, were determined by immunofluorescence analysis performed with Phospho-Histone H2A.X (Ser139, Cell Signaling Technology, USA) antibody or Histone H3K9me3 antibody (Active motif, USA). Cell nuclei were counterstained with the nuclear dye DAPI. Each condition was performed in *n* = 3, with *n* = 12 images taken per well (total 36 images) for analysis. Quantitative evaluation was performed on the images followed by an unbiased threshold-based analysis.

#### Analyses of cell senescence and epigenetic age

A stock solution of *Monarda didyma* L. extract (100 mg/mL) was prepared by dissolving the test compound in double-distilled water (ddH_2_O) and subsequent sterile filtration.

Primary human dermal fibroblasts, isolated from neonatal foreskin following circumcision surgery according to the previous protocol [38], were cultured at 37 °C and 5% CO_2_ in culture medium (DMEM with 10% FBS). Young fibroblasts (early passage, P7) were seeded (10^4^ cells/cm^2^) and grown for 24 h. The medium was then replaced with a culture medium, both with and without (control) the *Monarda didyma* L. extract solution (0.03 mg/mL) derived from the stock solution. The cells were incubated for 6 days, with the treatment renewed every 72 h. This process was repeated multiple times over several weeks in order to obtain different passages of aged fibroblasts, up to P17. At the start of the experiment (P7), at an intermediate timepoint (P13) and at the end (P17), 50 × 10^4^ cells, treated or not with the solution of *Monarda didyma* L., were frozen and stored for genomic DNA extraction and methylation analysis, and 20 × 10^4^ cells for analysis of SA-β-galactosidase activity. After thawing, young (P7) and aged (P17) fibroblasts were reseeded into 96-well plates (2 × 10^4^ cells/well) and cultured for 24 h in culture medium (negative control) and with *Monarda didyma* L. solution, and then, the cells were incubated for 72 h. Cells were then labeled with the SA-β-galactosidase probe and Hoechst solution 33258 (bis-benzimide, Sigma-Aldrich, USA) for cell nuclei. SA-β-galactosidase activity was assessed using the CellEvent™ Senescence Green Detection kit (Invitrogen, USA) according to the manufacturer’s instructions, and images of cells were obtained using a fluorescence microscope (INCell Analyzer™, GE Healthcare, USA). The fluorescent signal intensity was quantified using integration of numerical data with the Developer Toolbox 1.5 software (GE Healthcare) and normalized to the total number of cells. Methylation analyses were performed using Illumina EPIC methylation arrays (Illumina, USA) on DNA extracted from cells using the NucleoSpin® Tissue kit (Macherey–Nagel) according to the manufacturer’s instructions at P7, P13, and P17. DNAmAge was computed using the Horvath “Skin and Blood Clock” based on the analysis of methylation levels at specific sets of CpG sites [39]. Experiments were performed in *n* = 3 replicates per condition and timepoint. Microscopy analysis was performed on 5 images per well (*n* = 15 images for each condition and timepoint).

#### Analysis of endothelial function

Primary human umbilical vein endothelial cells (HUVEC, Promocell, Germany) were cultured in endothelial cell growth media (C-22010, Promocell), and confluent cells were treated with *Monarda didyma* L. extract solution at different concentrations (0.02, 0.2, and 2 mg/mL) and without (control) and with the positive [i.e., quercetin 0.015 mg/mL (antioxidant) and dexamethasone 0.02 mg/mL (anti-inflammatory)] and the negative [i.e., bacterial lipopolysaccharides (LPS) 0.025 mg/mL (pro-inflammatory)] reference compounds for either 4 h or 24 h. Levels of monocyte chemoattractant protein-1 (MCP-1) and vascular cell adhesion molecule (VCAM) were also measured by means of ELISA (R&D Systems, USA) in cell culture after 24 h.

After 24 h, cells were loaded with 0.002 mg/mL dichlorodihydrofluorescein (DCFH) and oxidative stress was induced by repeated addition of 0.085 mg/mL H_2_O_2_ to the culture medium, at initial time point and after 15 min. The intracellular antioxidant stress was analyzed after 30 min by measuring the intracellular ROS using DCFH that is converted to a fluorescent dye while reacting with ROS. Experiments were performed in *n* = 3–4 replicates per condition.

#### Analysis of vascular permeability

Primary human dermal microvascular endothelial cells (HDMEC, Promocell, Germany) were seeded (10^4^ cells/cm^2^) and grown to a confluent monolayer in 0.4 μm pore size Transwell inserts, coated with gelatin on polyester (PET) membranes, in endothelial cell growth media supplemented with growth medium kit (C-22010, Promocell) for 48 h at 37 °C and 5% CO_2_. After, the monolayers were incubated with *Monarda didyma* L. extract solution (0.3 mg/mL) and without (control), and with the reference compound APC366 (0.25 mg/mL, Bio-Techne, USA) as positive control diluted in culture medium for 1 h, followed by the addition of human recombinant β-tryptase (10 pmol/mL, Promega, USA) for 18 h to induce vascular permeability. After the treatment, Transwell inserts were transferred into wells containing fresh culture medium. FITC-dextran solution (Merck Millipore, USA) was added to the upper chamber and plates were incubated for 20 min. Afterwards, 100 μL medium of the lower well was collected and transferred to opaque black 96 well plates in triplicates. Fluorescence intensity was measured using a plate reader with OD at 475 nm. Vascular permeability was determined as fluorescence intensity (relative fluorescent units -RFU) correlated to the amount of FITC-dextran that passed through the cell layer on the Transwell insert. Experiments were performed in *n* = 6 replicates per condition.

#### Statistical analysis

Statistical comparisons were performed using GraphPad Prism 8 software (GraphPad, USA) and all data are expressed as mean ± SEM. Comparisons among different groups were made using One-way ANOVA, when comparing more than 2 variables, with Holm-Sidak post hoc test, or Student’s *T*-test when comparing two groups for TL analysis; *p*-values < 0.05 were considered significant.

### Clinical trial

#### Study population

Study population comprised a total of 81 participants, aged 45–65 years. All participants were employed by the University of Padua and included in the research during their routine health assessments conducted at the Occupational Medicine Unit—Azienda Ospedale Università di Padova (AOUP). Inclusion criteria were being between 45 and 65 years of age and being able and willing to follow the procedures of the study protocol. The exclusion criteria were as follows: (a) presence of any medical disorder potentially interfering with the study [heavy depression, diabetes, active cancer, severe liver disease, heavy cardiovascular disease (e.g., stroke, heart attack), and gastrointestinal diseases/conditions (e.g., ulcerative colitis, Crohn’s disease, peptic ulcer disease, celiac disease)]; (b) chronic intake of medication/dietary supplements with impact on stress levels (psychologically and physiological); (c) consumption of any dietary/nutritional supplements or functional foods; (d) being smokers; (e) drug-, alcohol-, and medication abuses; (f) known HIV-infection and acute or chronic hepatitis B and C infection; (g) known allergies against the mint family (Lamiaceae), especially lemon balm (cedronella, melissa, citronella); (h) being pregnant, breast feeding or intention to become pregnant during the study; (i) participation in another clinical study within the last 4 weeks and concurrent participation in another clinical study; (l) blood donation within 4 weeks prior to study start (screening) or during study; (m) anticipating any planned changes in lifestyle for the duration of the study. Prior to enrollment, all participants were thoroughly informed of the purpose of the study and of the protocol to be followed during recruitment at the Occupational Medicine Unit—AOUP. At the enrollment (T0) all participants signed the informed consent form (ICF) and the personal data processing form, both written in accordance with established criteria of the Institutional Review Board (IRB) and the appropriate federal regulations to describe the study plan, procedures, and risks. The CONSORT Flow Diagram in Supplementary Materials provides a detailed summary of the enrollment, allocation, follow-up, and analysis of participants in the clinical trial.

#### Study design

This study was a randomized, placebo-controlled, double-blind, parallel design, monocentric clinical trial. The study design and protocol conformed to the tenets of the Declaration of Helsinki and were approved by the local Ethics Committee—University of Padova (5518/AO/22; Study Code: EMODISU) and registered in the database for Protocol Registration and Results System ClinicalTrials.gov PRS (NCT05399966, registration date: May 17, 2022. ID: BIOAGELAB202201).

All eligible subjects for study participation were enrolled during their routine health assessments conducted at the Occupational Medicine Unit—AOUP from February 28, 2023, to March 16, 2023. They were randomly assigned in two different groups: the intervention group with nutritional supplementation of *Monarda didyma* L. extract (G1) and placebo control group with placebo supplementation (G2). The coordinating center and the steering committee generated the two comparison groups using simple randomization with an equal allocation ratio. Subjects in the two groups were matched for age, sex, and education, to avoid possible confounding variables. More details on randomization were reported in Supplementary note 1.

Participants underwent clinical examination to collect anthropometric measurements (e.g., height, weight, waist circumference, blood pressure), an interview with structured questionnaires administered by trained interviewers, and collection of fasting blood [for laboratory tests including biological age analyses (primary outcomes), basic biochemistry and inflammation markers (secondary outcomes)], and salivary sample for cortisol analysis. Clinical examination and sample collection, to assess all primary and secondary outcomes, were performed at enrollment (T0) and at the follow-up (T1), which occurred from May 22, 2023, to June 7, 2023, after 12 weeks (84 days) of treatment with dietary supplementation (*Monarda didyma* L. extract or placebo capsules). Each participant was assigned a MiBand 7 (Xiaomi) wearable device to monitor physical parameters (e.g., heart rate (HR), sleep, steps) during the study period (from baseline (T0) to follow-up (T1)). Participants also completed a diary throughout the study to assess compliance and to further monitor their well-being, health status, stress levels and treatment effects.

A comprehensive adverse event (AE) monitoring protocol was in place, which included participant reports, research team observations, laboratory assessments, registration on VigiErbe (http://www.vigierbe.it)—the platform for notifying the Istituto Superiore di Sanità (Ministry of Health, Italy)—and direct notification to Mibelle AG Biochemistry (Buchs, Switzerland), the producer of the *Monarda didyma* L. extract and placebo capsules.

Primary outcomes include DNAmAge and LTL. Secondary outcomes include blood pressure; salivary cortisol; blood count; inflammatory markers [C-reactive protein (CRP); interleukin-6 (IL6)]; questionnaires on quality of life, stress level, anxiety, sleep patterns, general well-being; monitoring of physical parameters using the MiBand 7 watch (HR, sleep, steps); total cholesterol; low-density lipoprotein (LDL); high-density lipoprotein (HDL); triglycerides; glycemia; insulin; homeostasis model assessment (HOMA) Index; aspartate transaminase (AST); alanine transaminase (ALT); gamma glutamyl transferase (GGT); creatinine; waist circumference; weight; total cholesterol/HDL; LDL/HDL; estimated glomerular filtration rate (eGFR).

#### Supplementation protocol

The supplementation protocol, started on the day of enrollment, required participants in the intervention group (G1) to take one capsule of *Monarda didyma* L. extract (100 mg/day) daily, preferably at lunchtime with plenty of water. While the placebo control group (G2) was required to take one capsule of Maltodextrin (100 mg/day) daily, preferably at lunch and with plenty of water.

According to the protocol, the supplementation was continued for a period of 12 weeks (84 days).

The *Monarda didyma* L. extract and placebo were provided in capsules (hydroxypropyl methylcellulose) with the same shape and appearance to allow double-blind performance. The capsules were provided in plastic flasks (pill boxes) with 50 capsules per flask. Each bottle was numbered consecutively for each participant according to the randomization schedule, and each participant received the capsules in the appropriate flask.

It is noteworthy that the *Monarda didyma* L. extract is recognized as safe for human consumption under European regulations and is included in the Italian regulatory framework for botanicals in food supplements [40], underscoring its suitability for long-term dietary interventions.

#### Questionnaires

Each participant was characterized by a data collection form structured to collect information on demographic variables, lifestyle and lifetime smoking history, dietary habits, marital status, years of education, institutionalization, occupations, environmental and occupational exposure to genotoxic factors, body mass index (BMI), and factors associated with biomarkers of aging. In addition, quality of life was assessed using the standardized World Health Organization’s Quality of Life Assessment, BREF version (WHOQOL-BREF) questionnaire [41]; sleep quality and efficiency were measured using the self-reported and standardized Pittsburgh Sleep Quality Index (PSQI) questionnaire [42]; and work ability was assessed using the standardized Work Ability Index (WAI) questionnaire [43, 44]. All questionnaires and the data collection form were administered to each participant at recruitment (T0) and follow-up (T1). A global assessment test was also administered at the follow-up questionnaire to collect satisfaction and evaluations of the whole study.

#### Sample collection

At enrollment (T0) and at the follow-up (T1), whole blood samples were collected from the antecubital vein of each study participant. Blood samples collected at T0, as part of routine worker surveillance at the Unit of Occupational Medicine Unit-AOUP, were sent to the Laboratory Medicine Unit-AOUP for routine analysis of blood parameters, plus an additional aliquot of blood for non-routine analyses of CRP, IL-6, AST, ALT and insulin, and morning saliva samples collected in a Salivette device (SARSTEDT AG & Co, Nümbrecht, Germany) for salivary cortisol levels. Additional tubes (i.e., 1 K2EDTA and 1 Blood RNA PaxGene) were used by the Laboratory of Environmental Mutagenesis (Department of Cardiac, Thoracic, Vascular and Public Health Sciences—DCTV, University of Padua) to analyze the indicators of biological aging. Blood and morning saliva samples were also taken at the follow-up (T1) in the same way as the non-routine analyses.

#### Monitoring through the wearable device MiBand 7 watch

HR monitoring, sleep tracking, daily step count, and energy expenditure data during the study period were recorded using MiBand 7 wearable devices (Xiaomi) provided on loan by DCTV—University of Padova. Participants were asked not to remove the device during the study unless absolutely necessary and only for short periods. The devices provided continuous monitoring of heart rate, energy expenditure, and sleep profile throughout the study period to assess metabolic demands and exercise patterns. Calibration was performed by comparing the data obtained with laboratory tests and with questionnaires administered, including data on lifestyle, physical activity, life, and sleep quality.

#### Basic biochemistry, inflammatory markers, and salivary cortisol

Basic biochemistry markers [blood count, total cholesterol (mg/dL), LDL (mg/dL), HDL (mg/dL), triglycerides (mg/dL), glycemia (mg/dL), insulin (mcU/mL), HOMA index, total cholesterol/HDL, LDL/HDL], inflammatory markers [CRP (mg/L) and IL-6 (ng/L)], indicators of liver [AST (IU/L), ALT (IU/L), GGT (U/L)], and kidney function [eGFR (mL/min/1.73 m^2^), creatinine (mg/dL)], and salivary cortisol were analyzed according to Laboratory Medicine Unit instructions (AOUP).

#### DNA extraction

DNA was extracted from whole blood using the QIAmpDNAmini Kit (QIAGEN, Milan, Italy) on the QIAcube System (QIAGEN, Milan, Italy) for automated DNA purification as previously described [45]. DNA samples were quantified and checked for quality using the QIAexpert Quantification System (QIAGEN, Milan, Italy).

#### DNAmAge

DNAmAge was measured by analyzing the methylation levels of five specific markers (ELOVL2, C1orf122, KLF14, TRIM59, and FHL2) in genomic DNA using bisulfite conversion and Pyrosequencing® methodology on the PyroMark Q48 Autoprep (QIAGEN, Milan, Italy) as previously described [46, 47]. The resulting Pyrograms® generated by the instrument were automatically analyzed using the Pyromark Q48 Autoprep software (QIAGEN, Milan, Italy). The methylation levels of the methylated cytosines at the 5 CpG sites were expressed as percentage and then used to estimate biological aging (years) as previously reported [46, 47].

#### LTL

LTL was determined by quantitative real-time PCR as previously described [48–50]. Relative LTL is measured in the genomic DNA of the experimental samples by determining the ratio of telomeric repeat copy number (T) and single copy gene (S) to the T/S ratio of a reference sample pool.

#### Hematological age: a new outcome

Hematological age was measured using Klemera and Dubal’s method [51] within the R language and environment for statistical computing and graphics v. 4.3.1 (https://www.r-project.org/) using basic biochemistry parameters, such as hemoglobin (g/L), mean corpuscular volume—MCV (fL), mean corpuscular hemoglobin concentration—MCHC (g/L), red blood cell distribution width—RDW (%), neutrophils (%), basophils (%), eGFR (mL/min/1.73m^2^), AST (U/L), ALT (U/L), total cholesterol (mg/dL), LDL (mg/dL), LDL/HDL, triglycerides (mg/dL), as well as BMI, systolic blood pressure (mmHg), diastolic blood pressure (mmHg) as clinical parameters, measured at baseline (T0), and follow-up (T1). Klemera and Doubal’s method first fits individual simple least-squares lines to each reference parameter as a function of chronological age. Hematological age is then estimated by minimizing the distance between these individual regression lines and the observed data points of the reference parameters within the space of all biochemistry and clinical parameters.

#### Sample size

The sample size per group was calculated for a two-time longitudinal study based on data obtained from our previous study [17] by using the StatsDirect software and R version 4.3.1. In particular, as telomere analysis represents the limiting analysis, considering a difference in LTL of − 0.20 (SD 0.29), 22 subjects are required to achieve 80% power to detect the association with an alpha error ≤ 5%. Therefore, although the number would be 22, we included 40 subjects for both the intervention and control groups to account for any discontinuation of participation in the study (dropout).

#### Statistical analysis

The study data were analyzed exploratively. All data collected, obtained and documented in this study were listed and summarized using descriptive statistics or frequency tables, as appropriate. Data were expressed as mean ± SD or number and percentage.

All statistical tests were performed two-sided using StatsDirect software and R 4.3.1. Efficacy endpoints were tested using an analysis of variance (ANOVA) and pairwise Student's *t*-test for each group, and Pearson chi-square test and Mann–Whitney *U* test between the intervention group and the placebo control group, considering the variable of interest (i.e., all primary and secondary outcomes). The effect size = mean difference divided by the population standard deviation, i.e., the normalized mean difference, was also calculated. Simple linear regression was evaluated in order to provide a measure of the strength of dependence between two variables. Furthermore, the Bland–Altman method [52] was used to determine the agreement between the MiBand 7 wearable device and PSQI questionnaire for sleep parameters. Each comparison was treated as an independent research question, with *p* ≤ 0.05 considered statistically significant.

#### Movement and sleep indexes (MI and SI)

MI was calculated using the R v 4.3.1 environment, considering the physical activity variables steps (n), distance (m), and run distance (m) recorded by the MiBand 7 wearable devices during the study period (from T0 to T1). All three variables were first normalized to the interval [0,1] using ranks. The MI is the weighted average of these normalized values with weights 0.4 (steps), 0.35 (distance), and 0.25 (run distance).

SI was calculated using the R v. 4.3.1 environment considering the data on total sleep time (min) and rapid eye movement (REM) time (min) recorded by MiBand 7 wearable devices during the study period (from T0 to T1). Both variables were first normalized to the interval [0,1] using ranks. The SI is the simple average of these two normalized values with weights 0.5 (total sleep time) and 0.5 (REM time).

## Results

### In vitro studies on *Monarda didyma* L. extract

This section presents findings from targeted in vitro studies investigating the cellular and molecular effects of *Monarda didyma L.* extract, chosen for its potential to modulate key hallmarks of aging. The analyses encompass protein oxidation, telomere length dynamics, DNA damage-induced aging, cellular senescence, epigenetic age, endothelial function, and vascular permeability. These assays were chosen for their biological plausibility and relevance to age-related processes and pathologies. The extract’s potent antioxidant properties, along with its capacity to support cellular repair mechanisms, preserve endothelial integrity, and mitigate vascular dysfunction, form a compelling basis for its potential geroprotective effects.

#### Analysis of protein oxidation

**Fig. 1:**
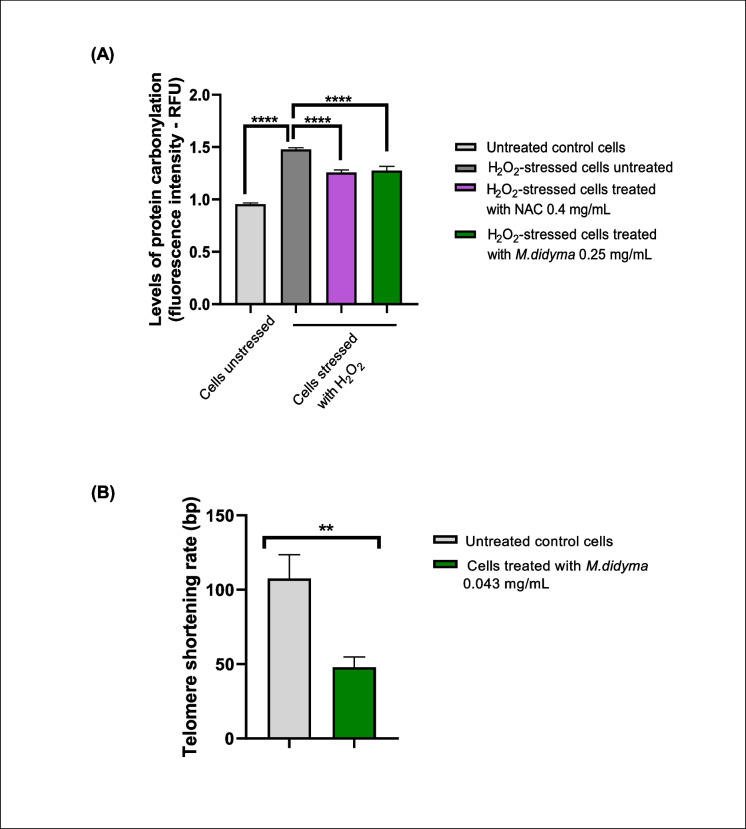
In vitro studies on *Monarda didyma* L. extract: protein oxidation and telomere shortening rate (**A**, **B**). **A**, effect of *Monarda didyma* L. extract solution and NAC, which is used as a positive control, on protein carbonylation levels in fibroblasts. The quantification of protein carbonylation levels is reported as relative fluorescence unit (RFU) normalized to cells unstressed. **B**, effect of *Monarda didyma* L. extract solution on telomere length in fibroblasts measured as telomere shortening rate (base pairs, bp). One-way ANOVA was used for statistical analysis of protein carbonylation levels, while Student's *T*-test was used for statistical analysis of telomere shortening rate; **, *p* < 0.01; ****, *p* < 0.0001

As shown in Fig. 1A, cells exposed to H_2_O_2_ exhibited a significant increase in carbonylated (oxidized) proteins compared to untreated cells (*p* < 0.0001). However, treatment with *Monarda didyma* L. extract or NAC, a potent antioxidant used as a positive control, significantly reduced protein carbonylation levels in H_2_O_2_-stressed cells (*p* < 0.0001). The extent of this reduction was comparable between the two treatments (*p* = 0.9563).

#### TL

As shown in Fig. 1B, cells treated with *Monarda didyma* L. extract for 6 weeks showed a significant reduction in the rate of telomere shortening compared to untreated control cells (*p* = 0.0091).

#### DNA damage-induced aging

**Fig. 2:**
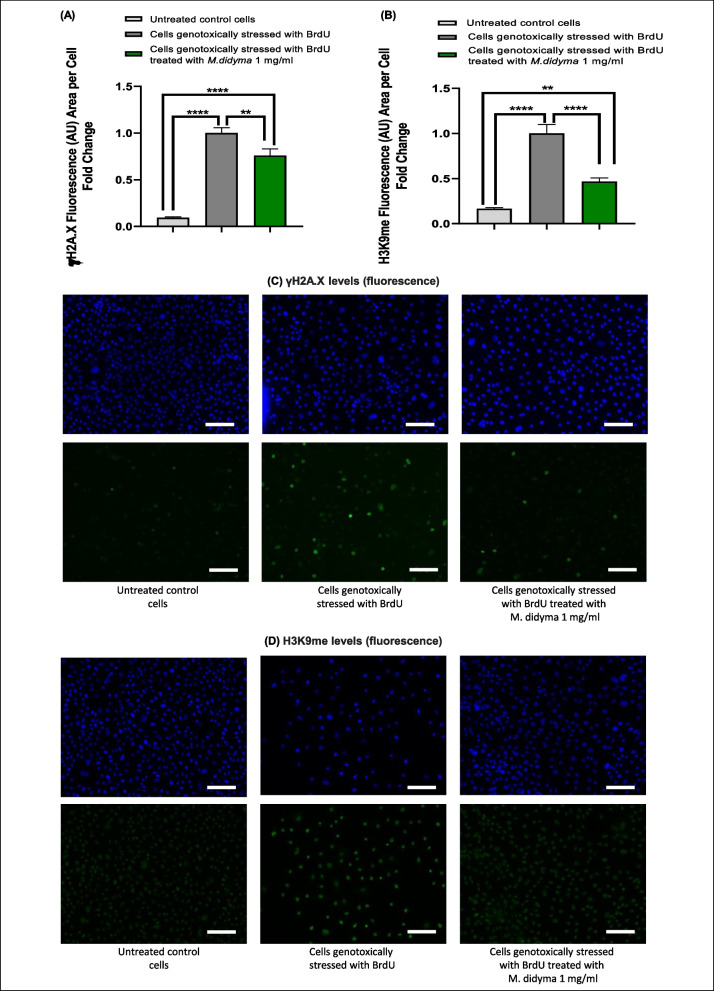
In vitro studies on *Monarda didyma* L. extract: DNA damage-induced aging (**A**, **B**, **C**, **D**). **A**, **C**, effect of *Monarda didyma* L. extract solution on γH2A.X levels in human epidermal keratinocytes. The quantification of γH2A.X levels is reported as fluorescence (AU) area per cell fold change (**A**) and as Alexa 488 mean fluorescence intensity (green) (**C**). Cell nuclei are stained with DAPI (blue) and the images present a scale bar of 50 μm, indicated by the white bar. **B**, **D**, effect of *Monarda didyma* L. extract solution on H3K9 levels in human epidermal keratinocytes. The quantification of H3K9 levels is reported as fluorescence (AU) area per cell fold change (**B**) and as Alexa 488 mean fluorescence intensity (green) (**D**). Cell nuclei are stained with DAPI (blue) and the images present a scale bar of 50 μm, indicated by the white bar. Data presented in **A**, **B**, **C**, and **D** are reported as mean ± SEM. One-way ANOVA was used for statistical analysis. **, *p* < 0.01; ****, *p* < 0.0001

Figure 2 A and C illustrate a marked increase of γH2A.X levels in cells genotoxically stressed with BrdU (*p* < 0.0001), both in the presence and absence of *Monarda didyma* L. extract, compared to untreated control cells. However, cells treated with *Monarda didyma* L. extract showed significantly reduced γH2A.X signal compared to stressed control cells (*p* = 0.0048). Similarly, Fig. 2B and D depict a substantial increase of H3K9me3 levels in cells genotoxically stressed with BrdU (both treated with the *Monarda didyma* L. extract (*p* = 0.0026) and without (*p* < 0.0001)) when compared to untreated control cells. Nonetheless, cells treated with *Monarda didyma* L. extract presented significantly lower H3K9me3 levels compared to stressed control cells (*p* < 0.0001).

#### Cell senescence and epigenetic age

**Fig. 3:**
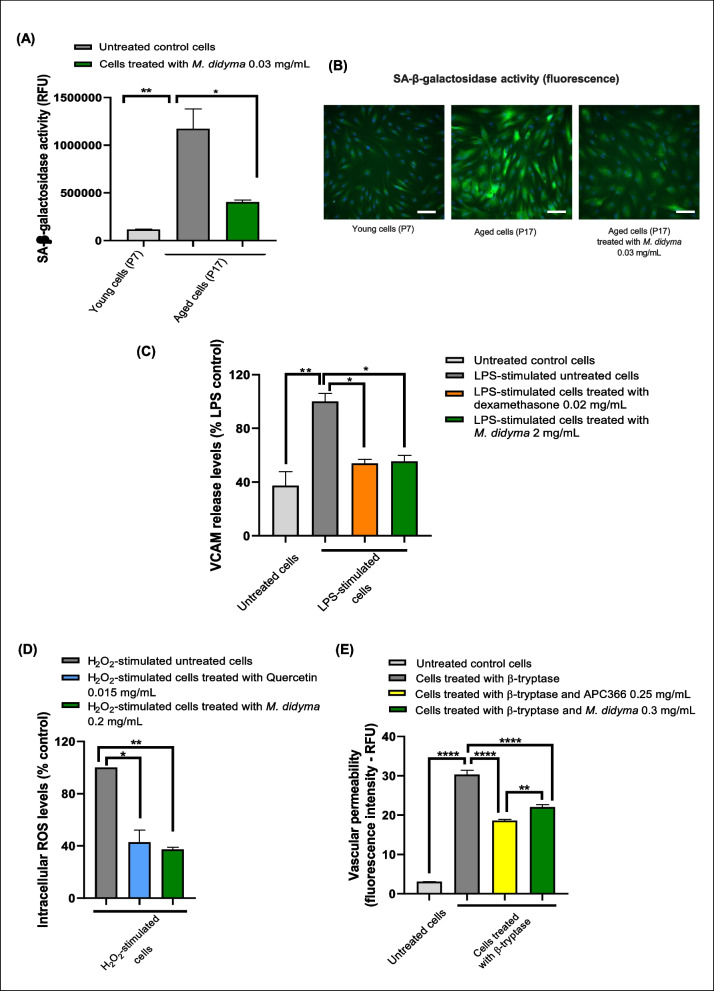
In vitro studies on *Monarda didyma* L. extract: cell senescence, endothelial function, and vascular permeability. **A**, **B**, effect of *Monarda didyma* L. extract solution on SA-β-galactosidase activity in aged fibroblasts. The quantification of SA-β-galactosidase activity is reported as relative fluorescence unit (RFU) (**A**) and as Alexa Fluor 488/FITC mean fluorescence intensity (green) (**B**). The images present a scale bar of 50 μm, indicated by white bar. Cell nuclei are stained with Hoechst (blue). **C**, effect of *Monarda didyma* L. extract solution and dexamethasone, used as a positive control, on VCAM levels in primary human umbilical vein endothelial cells (HUVEC). The quantification of VCAM levels is reported compared to control LPS in percentage (%). **D**, effect of *Monarda didyma* L. extract solution and Quercetin, used as a positive control, on intracellular ROS levels in HUVEC cells. The quantification of intracellular ROS levels is reported compared to control in percentage (%). **E**, effect of *Monarda didyma* L. extract solution and APC366, used as a positive control, on vascular permeability in human dermal microvascular endothelial cells. The quantification of vascular permeability is reported as relative fluorescence unit (RFU). Data presented in **A**, **B**, **C**, **D**, and **E** are reported as mean ± SEM. One-way ANOVA was used for statistical analyses. *, *p* < 0.05; **, *p* < 0.01; ****, *p* < 0.0001

Figure 3 A and B demonstrate a significant reduction in SA-β-galactosidase activity in aged cells treated with *Monarda didyma* L. extract compared to untreated aged control cells (*p* = 0.0214). The graph also confirms the expected increase in SA-β-galactosidase activity in aged control cells compared to younger ones (*p* = 0.0071), validating our analysis. Supplementary Fig. 1 further shows a reduction in the epigenetic age of aged cells treated with *Monarda didyma* L. extract, showing an approximate decrease of 12 years compared to untreated aged controls, although this difference was not statistically significant (*p* = 0.227). The graph also shows a progressive increase in biological age with successive cell passages, from young cells (P7) to middle-aged cells (P13, *p* = 0.0177) and finally to aged untreated control cells (P17, *p* = 0.0769).

#### Endothelial function

Figure 3 C shows that cells stimulated with bacterial LPS exhibited a significant increase in the release of VCAM levels compared to untreated control cells (*p* = 0.0087). LPS-stimulated cells treated with *Monarda didyma* L. showed a significant reduction in VCAM release after 24 h compared to LPS-stimulated untreated cells (*p* = 0.0289). This effect was similar to that observed in LPS-stimulated cells treated with dexamethasone, a positive anti-inflammatory control (*p* = 0.0259). Supplementary Fig. 2 indicates an increase in MCP-1 release in LPS-stimulated cells compared to untreated control cells (*p* < 0.0001). However, LPS-stimulated cells treated with *Monarda didyma* L. extract or dexamethasone did not show a significant reduction in MCP-1 levels after 24 h compared to LPS-stimulated cells untreated (*p* > 0.05). Finally, Fig. 3D illustrates that H_2_O_2_-stimulated cells treated with *Monarda didyma* L. extract solution exhibited a significant decrease in intracellular ROS levels after 24 h compared to H_2_O_2_-stimulated untreated cells (*p* = 0.0079), a reduction comparable to that of H_2_O_2_-stimulated cells treated with quercetin as a positive control (*p* = 0.0103).

#### Vascular permeability

Microvascular endothelial cells treated with β-tryptase showed a significant increase in permeability, as indicated by a strong fluorescence signal compared to untreated control cells (*p* < 0.0001, Fig. 3E). However, treatment with *Monarda didyma* L. extract resulted in a markedly lower fluorescence signal, indicating reduced permeability, when compared to cells treated with β-tryptase alone (*p* < 0.0001). Similarly, treatment with the positive control, the tryptase inhibitor APC366, also showed reduced permeability (*p* < 0.0001), although their fluorescence signal was still higher than that observed in cells treated with both β-tryptase and *Monarda didyma* L. extract (*p* = 0.0059).

### Clinical trial

This section bridges preclinical findings with clinical application, evaluating the effects of *Monarda didyma L.* extract on key markers of biological aging, including LTL and DNAmAge, as well as quality of life, physical activity, and biochemical parameters. These outcomes were selected for their relevance to aging processes and their ability to provide a multidimensional assessment of the extract’s geroprotective potential. By building on the mechanistic insights from in vitro studies, the clinical trial offers a holistic perspective on the extract’s efficacy in modulating age-related biomarkers and improving overall health.

#### Study population

The study population consists of *N* = 81 participants randomly assigned to intervention group (G1, *N* = 40) and control group (G2, *N* = 41). Table 1 summarizes the characteristics of study participants at recruitment (T0) for group G1 and group G2, including demographic variables, marital status, educational level, employment history, lifestyle, and information on SARS-CoV-2 infection prior to the clinical trial. The same characteristics are summarized for all participants (*N* = 81) in Supplementary Table 1. No significant adverse effects were reported by study participants.

**Table 1:** Study population characteristics of G1 group (*N* = 40) and G2 group (*N* = 41) at enrollment visit (T0)

Variable	G1 (*N* = 40)			G2 (*N* = 41)		
	*Mean* ± *SD*	*N subjects*	*%*	*Mean* ± *SD*	*N subjects*	*%*
Age (years)	55.23 ± 4.15			56.34 ± 5.01		
Gender:						
M		20	50.00		20	48.78
F		20	50.00		21	51.22
Marital status:						
Not married		8	20.00		3	7.32
Married		21	52.50		29	70.73
Cohabiting		6	15.00		7	17.07
Divorced		4	10.00		2	4.88
Widower		1	2.50		0	0.00
Years of education (years):	18.60 ± 4.25			18.39 ± 4.15		
Lower (high school diploma)	12.0 ± 1.93	8	20.00	12.58 ± 1.0	12	29.27
Medium (bachelor’s degree, master’s degree)	17.46 ± 1.27	13	32.50	17.67 ± 0.82	6	14.64
High (postgraduation degree, PhD)	22.16 ± 1.71	19	47.50	21.61 ± 1.12	23	56.10
Weight (kg)	73.23 ± 12.56			69.55 ± 13.29		
Height (m)	1.71 ± 0.07			1.69 ± 0.08		
Abdominal circumference (cm)	90.51 ± 12.05			87.28 ± 11.51		
BMI (kg/m^2^):	24.88 ± 2.95			24.12 ± 3.38		
Underweight ($<$ 18.5)		1	2.50		1	2.44
Normal range ($\ge$ 18.5; < 25.0)		20	50.00		24	58.54
Overweight ($\ge$ 25.0; < 30.0)		17	42.50		15	36.59
Obese (≥ 30.0)		2	5.00		1	2.44
Systolic blood pressure (mmHg)	126.05 ± 16.87			121.29 ± 15.14		
Diastolic blood pressure (mmHg)	82.58 ± 9.94			79.59 ± 8.60		
***Employment anamnesis***						
Professional position:						
Professor/researcher/doctor		17	42.50		16	39.02
Laboratory or informatic technician		10	25.00		12	29.27
Administrative technician or librarian		13	32.50		13	31.71
Years of work in the current job (years)	17.00 ± 9.58			17.98 ± 10.42		
Performance of night shifts		1	2.50		1	2.44
Work risks:						
None		2	5.00		2	4.88
Chemical		20	50.00		14	34.15
Biological		17	42.50		14	34.15
VDT		15	37.50		19	46.34
Other		1	2.50		2	4.88
***Lifestyle***						
Tobacco habit:						
Ex-smoker		10	25.00		11	26.83
Non-smoker		30	75.00		30	73.17
Exposure to second-hand smoke		2	5.00		3	7.32
Pack/years [(cigarettes/20) per years of ex smoking]	4.26 ± 4.68			6.82 ± 6.11		
Alcohol consumption:						
Daily (05 or 1 UA)		16	40.00		18	43.90
Occasionally		12	30.00		16	39.02
Non-consumer		12	30.00		7	17.07
Physical activity		32	80.00		32	78.05
Type of physical activity:						
None		8	20.00		9	21.95
Walking		9	22.50		12	29.27
Running		6	15.00		6	14.63
Cycling		7	17.50		4	9.76
Gym/yoga		16	40.00		11	26.83
Other sports (paddel, swimming, rowing, tennis, dancing, football, etc..)		5	12.50		8	19.51
Years of physical activity:	17.77 ± 13.59			14.54 ± 13.65		
0–3 years		6	15.00		7	17.07
3–10 years		9	22.50		12	29.27
> 10 years		17	42.50		13	31.71
Frequency of vegetable meals (n/week)	10.13 ± 3.45			10.32 ± 2.64		
Frequency of fruit meals (n/week)	8.40 ± 3.76			9.12 ± 4.43		
Indoor pollution—heating:						
Wood stove		2	5.00		4	9.76
Pellet stove		0	0.00		3	7.32
Alcohol/bioethanol stove		1	2.50		1	2.44
Gas		39	97.50		40	97.56
Photovoltaic panels		1	2.50		3	7.32
Fireplace in the house (wood and pellet)		5	12.50		6	14.63
Living area:						
Non-urban/rural area		8	20.00		8	19.51
Urban/peripheral area		32	80.00		33	80.49
House close to industrial settlements		6	15.00		6	14.63
Traffic in the living area:						
Continuous intense for a good part of the day		11	27.50		13	31.71
Intermittent intense		16	40.00		16	39.02
Scarce or absent		13	32.50		12	29.27
Means of transport usually used:						
On foot		15	37.50		14	34.35
Bike		24	60.00		15	36.59
Car and motorbike		24	60.00		25	60.98
Bus/tram/train		6	15.00		5	12.20
Total travel time:						
Up to 30 min		6	15.00		9	21.95
30–60 min		14	35.00		20	48.78
> 60 min		20	50.00		12	29.27
***COVID-19 infection before the clinical trial:***						
None		10	25.00		16	39.02
Once		27	67.50		20	48.78
Reinfection		3	7.50		5	12.20
Vaccine doses:						
0		0	0.00		1	2.44
1		1	2.50		0	0.00
2		2	5.00		3	7.32
3		24	60.00		22	53.66
4		13	32.50		15	36.59
***Biological age markers***	***Mean* ± *SD G1 (N* = *40)***			***Mean* ± *SD G2 (N* = *41)***		
DNAmAge	46.23 ± 4.92			47.85 ± 5.36		
LTL	1.49 ± 0.35			1.47 ± 0.37		
Hematological age	55.83 ± 8.55			55.61 ± 8.92		

#### Basic biochemistry parameters before and after treatment

Table 2 shows comparisons of the basic hematochemical parameters, biomarkers of liver and kidney function and inflammation examined in the study population before (T0) and after (T1) treatment in G1 (*N* = 40) and G2 (*N* = 41) participant’s groups. Variations were detected between T0 and T1 or between the two groups (G1 and G2). In particular, G2 group showed an increase in neutrophils (*p* = 0.0237), monocytes (*p* = 0.0571) and RDW (*p* = 0.0161), and a decrease in hematocrit (*p* = 0.0125) and MCV (*p* = 0.0008) after treatment. Furthermore, a decrease in creatinine (*p* = 0.0188) and an increase in eGFR (*p* = 0.0405) among the renal function parameters and in IL-6 levels (*p* = 0.0063) among inflammation parameters were also observed in the G2 group. Decreased levels of hemoglobin (G1, *p* = 0.0225; G2, *p* = 0.0416), glycemia (G1, *p* = 0.0004; G2, *p* = 0.0051) and salivary cortisol (G1, *p* = 0.0147; G2, *p* = 0.0078) after treatment were detected in both G1 and G2 groups. In addition, the G2 group showed elevated levels of HDL (*p* = 0.042), and lower levels of total cholesterol/HDL (*p* = 0.0169) and LDL/HDL cholesterol ratio (*p* = 0.0282) after treatment compared to the G1 group.

**Table 2:** Basic biochemistry parameters measured before and after treatment in G1 (*N* = 40) and G2 (*N* = 41)

Basic biochemistry parameters	*G1*							*G2*							*G1 vs G2 (T1)* *Mann–Whitney U test*
	T0 Mean ± SD	T0 N subjects out of range	T0 % subjects out of range	T1 Mean ± SD	T1 N subjects out of range	T1 % subjects out of range	Paired *t* test	T0 Mean ± SD	T0 N subjects out of range	T0 % subjects out of range	T1 Mean ± SD	T1 N subjects out of range	T1 % subjects out of range	Paired t test	
Leukocytes (10^9^/L) (range: 4.40–11.00)	5.82 ± 1.07	3	7.5	5.84 ± 1.18	1	2.5	0.8487	5.69 ± 1.21	4	9.76	5.98 ± 1.55	4	9.76	0.0929	0.7334
Erythrocytes (10^12^/L) (range: F 4.31–5.10; M 4.50–5.90)	4.84 ± 0.57	4	10	4.80 ± 0.56	7	17.5	0.1663	4.73 ± 0.42	9	21.95	4.68 ± 0.38	5	12.20	0.1738	0.6068
Hemoglobin (g/L) (range: F 123–153; M 140–175)	146.3 ± 12.89	1	2.5	144.1 ± 12.59	3	7.5	**0.0225**	144.7 ± 11.14	1	2.44	142.7 ± 10.73	2	4.88	**0.0416**	0.7015
Hematocrit (L/L) (range: F 0.36–0.45; M 0.41–0.50)	0.44 ± 0.03	0	0.0	0.43 ± 0.03	3	7.5	0.0879	0.44 ± 0.03	4	9.76	0.43 ± 0.03	2	4.88	**0.0125**	0.5679
Platelet count (10^9^/L) (range: 150–450)	260.1 ± 50.94	1	2.5	248.9 ± 52.33	1	2.5	0.0929	244.2 ± 55.50	1	2.44	237.6 ± 54.28	1	2.44	0.1043	0.3407
Neutrophils (10^9^/L) (range: 1.80–7.80)	3.11 ± 0.83	1	2.5	3.16 ± 0.88	1	2.5	0.6554	3.11 ± 0.98	4	9.76	3.41 ± 1.17	2	4.88	**0.0237**	0.3985
Lymphocytes (10^9^/L) (range: 1.10–4.80)	2.02 ± 0.48	1	2.5	1.99 ± 0.53	0	0.0	0.6168	1.89 ± 0.45	3	7.32	1.87 ± 0.54	2	4.88	0.7613	0.2535
Monocytes (10^9^/L) (range: 0.20–0.96)	0.49 ± 0.12	0	0.0	0.49 ± 0.13	0	0.0	0.7277	0.49 ± 0.13	1	2.44	0.52 ± 0.16	2	4.88	0.0571	0.5331
Eosinophils (10^9^/L) (range: 0.00–0.50)	0.15 ± 0.09	1	2.5	0.16 ± 0.08	0	0.0	0.8442	0.16 ± 0.09	0	0.0	0.16 ± 0.08	0	0.0	0.7110	0.8158
Basophils (10^9^/L) (range: 0.00–0.20)	0.04 ± 0.02	0	0.0	0.05 ± 0.02	0	0.0	0.4195	0.04 ± 0.01	0	0.0	0.04 ± 0.02	0	0.0	> 0.9999	0.1112
Mean corpuscular volume—MCV (fL) (range: 80.0–96.0)	90.81 ± 5.47	4	10	90.54 ± 5.58	2	5.0	0.1616	92.19 ± 4.45	6	14.63	91.40 ± 4.52	6	14.63	**0.0008**	0.9831
MCH (pg) (range: 26.0–33.0)	30.4 ± 2.24	1	2.5	30.18 ± 2.24	1	2.5	**0.0041**	30.66 ± 1.60	2	4.88	30.52 ± 1.66	2	4.88	0.0586	0.7438
MCHC (g/L) (range: 320–360)	334.7 ± 12.43	2	5.0	333.1 ± 11.81	5	12.5	0.1860	332.6 ± 7.74	1	2.44	333.9 ± 8.68	2	4.88	0.3149	0.9794
Red cells distribuition width—RDW (%) (range: 11.2–15.6)	12.82 ± 1.15	1	2.5	12.92 ± 1.21	1	2.5	0.0556	12.72 ± 0.58	0	0.0	12.91 ± 0.60	0	0.0	**0.0161**	0.3467
Glycemia (mmol/L) (range: 3.7–5.6)	5.09 ± 0.53	5	12.5	4.78 ± 0.59	2	5.0	**0.0004**	5.04 ± 0.49	2	4.88	4.84 ± 0.46	1	2.44	**0.0051**	0.2384
Cholesterol (mmol/L) (desirable < 5.18)	5.35 ± 0.85			5.27 ± 0.83			0.4072	5.17 ± 0.87			5.20 ± 0.87			0.7333	0.6844
Borderline 5.18–6.19		15	37.5		17	42.5			14	34.15		14	34.15		
High > 6.19		5	12.5		5	12.5			5	12.20		5	12.20		
Triglycerides (mmol/L) (desirable < 1.69)	1.16 ± 0.78	5	12.5	1.21 ± 0.95	6	15	0.6365	0.90 ± 0.32	1	2.44	0.86 ± 0.29	0	0.0	0.3383	0.2362
High-density lipoproteins—HDL (mmol/L) (desirable > 1.04)	1.50 ± 0.37	6	15	1.52 ± 0.43	5	12.5	0.4986	1.70 ± 0.48	5	12.20	1.73 ± 0.49	3	7.32	0.3874	**0.0420**
Low-density lipoproteins—LDL (mmol/L)	3.48 ± 0.78			3.40 ± 0.82			0.3465	3.24 ± 0.80			3.25 ± 0.81			0.8807	0.3038
Borderline 3.34–4.12		16	40		10	25			15	36.59		12	29.27		
High > 4.12		8	20		5	12.5			4	9.76		6	14.63		
Total cholesterol/HDL (desirable < 5)	3.79 ± 1.10	5	12.5	3.75 ± 1.20	8	20	0.6036	3.24 ± 0.97	3	7.32	3.23 ± 1.06	3	7.32	0.8052	**0.0169**
LDL/HDL cholesterol (desirable < 3)	2.43 ± 0.81	9	22.5	2.42 ± 0.90	10	25	0.9976	2.11 ± 0.90	7	17.07	2.07 ± 0.94	6	14.63	0.9218	**0.0282**
Insulin (mU/L) (range: 3.2–16.3)	8.35 ± 5.44	5	12.5	7.60 ± 4.86	8	20	0.2014	7.05 ± 4.36	5	12.20	6.72 ± 3.06	4	9.76	0.6121	0.7979
Lower than 3.2		2	5		4	10			4	9.76		4	9.76		
Higher than 16.3		3	7.5		4	10			1	2.44		0	0.0		
HOMA index (desirable < 2.5)	1.91 ± 1.31	4	10	1.65 ± 1.15	7	17.5	0.0777	1.61 ± 1.11	3	7.32	1.46 ± 0.71	3	7.32	0.3494	0.9794
***Liver function***															
Aspartate aminotransferase—AST (U/L) (range: F 10–35; M 10–45)	22.67 ± 4.70	0	0.0	22.9 ± 5.82	1	2.5	0.7589	23.05 ± 4.32	0	0.0	23.32 ± 4.93	0	0.0	0.6504	0.7256
Alanine aminotransferase – ALT (U/L) (range: F 7–35; M 10–50)	21.92 ± 7.78	2	5.0	20.4 ± 9.71	2	5.0	0.1122	22.76 ± 8.64	0	0.0	21.07 ± 8.73	3	7.32	0.1051	0.3568
Gamma glutamyl transferase—GGT (U/L) (range: F 3–45; M 3–65)	18.75 ± 17.04	1	2.5	20.02 ± 18.77	1	2.5	0.1449	19.88 ± 16.04	2	4.88	20.76 ± 19.84	1	2.44	0.5136	0.9008
***Kidney function***															
Estimated glomerular filtration rate—eGFR (mL/min/1.73 m^2^) (desirable > 90)	89.05 ± 11.17	20	50	90.35 ± 9.32	17	42.5	0.3429	87.22 ± 10.20	24	58.54	89.78 ± 12.11	18	43.90	**0.0405**	0.9671
Creatinine (μmol/L) (range: F 45–84; M 59–104)	75.52 ± 12.96	1	2.5	74.12 ± 10.19	0	0.0	0.2302	76.78 ± 13.33	0	0.0	74.10 ± 14.56	4	9.76	**0.0188**	0.7797
***Inflammation***															
C-reactive protein—CRP (mg/L) (range: 0.00–5.00)	1.11 ± 0.97	1	2.5	1.10 ± 0.82	0	0.0	0.9157	1.68 ± 3.28	3	7.32	1.07 ± 0.78	0	0.0	0.2510	0.5377
Interleukin-6—IL-6 (ng/L) (range: 3.0–7.0)	1.58 ± 3.00	2	5.0	1.50 ± 1.07	0	0.0	0.8634	0.71 ± 0.65	0	0.0	1.28 ± 1.06	0	0.0	**0.0063**	0.2888
Salivary cortisol (nmol/L) (range: 3.0–21.1)	5.91 ± 4.26	0	0.0	4.43 ± 2.33	0	0.0	**0.0147**	4.68 ± 2.42	0	0.0	3.85 ± 1.85	0	0.0	**0.0078**	0.1522

Bold character is displayed only for significant values (*p*<0.05); *T*-test is performed for paired continuous variables; Mann-Whitney *U* test is performed for unpaired continuous variables

#### Biological age markers ˗ LTL and DNAmAge ˗ before and after treatment

**Fig. 4:**
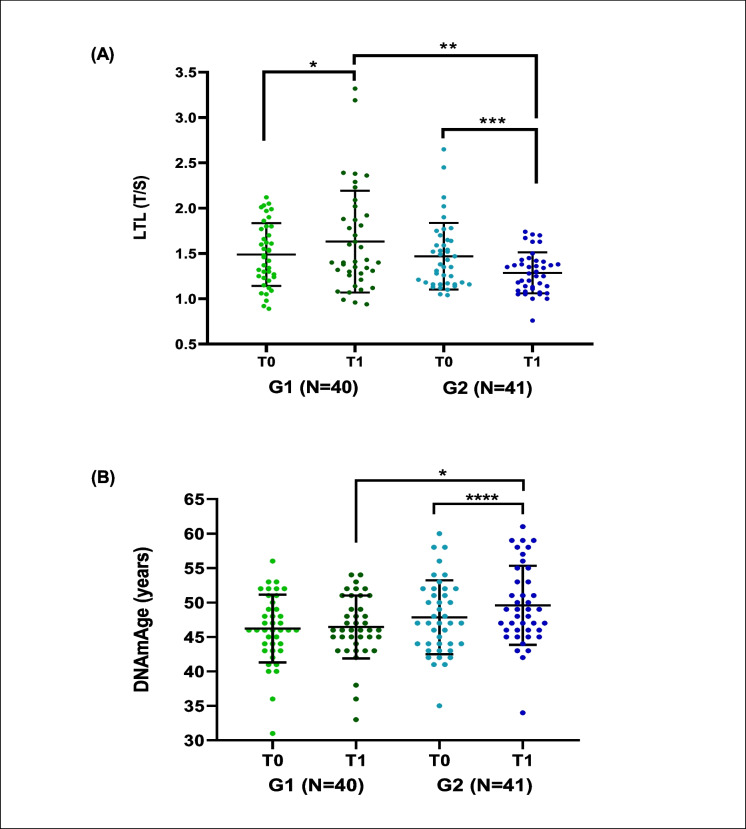
Clinical trial on *Monarda didyma* L. extract: LTL and DNAmAge in G1 (intervention group, *N* = 40) and G2 (placebo group, *N* = 41) before and after treatment (**A**, **B**). **A**, LTL in G1 and G2 groups before and after treatment with *Monarda didyma* L. or placebo, respectively. LTL is reported as T/S before (T0) and after (T1) treatment in the G1 group (green) and in the G2 group (blue). **B**, DNAmAge in G1 and G2 groups before and after treatment with *Monarda didyma* L. or placebo, respectively. DNAmAge is reported in years before (T0) and after (T1) treatment in the G1 group (green) and in the G2 group (blue). Data in **A** and **B** are reported as mean ± SD. Student’s *T*-test and Mann–Whitney *U* test were used for statistical analyses; *, *p* < 0.05; **, *p* < 0.01; ***, *p* < 0.001; ****, *p* < 0.0001

The marker of mitotic cellular aging, LTL, depicted in Fig. 4A, exhibited an increase after treatment in the G1 group (*p* = 0.0537), whereas it showed a more significant decrease in the G2 group (*p* = 0.0006). When comparing post-treatment LTL values between the groups, it was found that the LTL in G1 was significantly longer than the LTL in G2 (*p* = 0.0041), while no difference was observed at baseline (*p* = 0.0578).

DNAmAge, representing non-mitotic cellular aging shown in Fig. 4B, remained stable after treatment in the G1 group (*p* = 0.4522), whereas it exhibited a significant increase in the G2 group (*p* < 0.0001). When comparing post-treatment DNAmAge values between the G1 and G2 groups, it was evident that the DNAmAge of G2 was significantly higher than that of G1 (*p* = 0.0162), whereas no difference was observed at baseline (*p* = 0.2787).

**Fig. 5:**
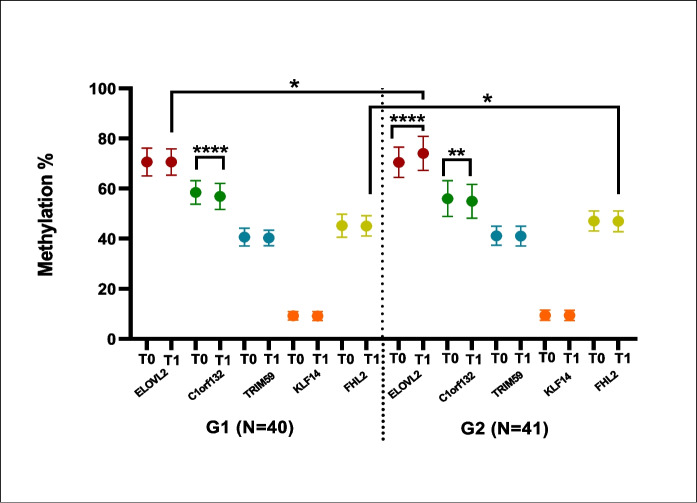
Clinical trial on *Monarda didyma* L. extract: DNA methylation status at the CpG sites of five genes analyzed. DNA methylation status at the CpG sites of each of the five genes (ELOVL2, C1orf132, KLF14, TRIM59, and FHL2) analyzed for DNAmAge determination, reported as methylation %, before (T0) and after (T1) treatment of the G1 (intervention group, *N* = 40) and G2 (placebo group, *N* = 41). Data presented are reported as mean ± SD. Student’s *T*-test and Mann–Whitney *U* test were used for statistical analyses; *, *p* < 0.05

The DNA methylation status at the CpG sites of each of the five genes analyzed for DNAmAge determination (ELOVL2, C1orf132, KLF14, TRIM59 and FHL2) before and after treatment of the G1 and G2 groups is shown in Fig. 5 and in Supplementary Table 2. Both G1 (*p* = 0.0001) and G2 (*p* = 0.0104) groups showed a significant decrease in the methylation pattern of C1orf132 after treatment. However, the placebo-treated G2 group showed an increase in the methylation of ELOVL2 (*p* < 0.0001) which was not observed in the G1 group. Furthermore, as illustrated in Fig. 5, when comparing the post-treatment methylation pattern of these five markers between the G1 and G2 groups, hypermethylation of ELOVL2 and FHL2 was observed in G2 compared to G1 (*p* = 0.0452).

#### Hematological age before and after treatment in G1 and G2

Hematological age, a newly developed marker of aging reported in Supplementary Table 3, displayed no significant changes in either the G1 (*p* = 0.4965) or G2 groups (*p* = 0.5464), nor between the G1 and G2 groups before (*p* = 0.8454) or after treatment (*p* = 0.3677).

#### Data from questionnaires and MiBand 7 watch

**Fig. 6:**
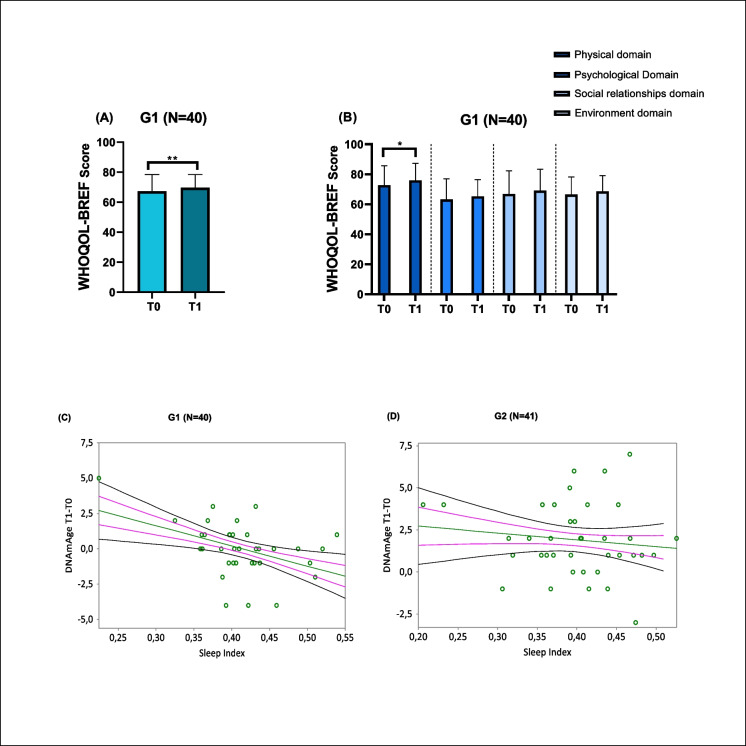
Clinical trial on *Monarda didyma* L. extract: data from questionnaires and Sleep Index derived by MiBand 7 data. **A**, **B**, WHOQOL-BREF questionnaire on quality of life and details of the different domains making up the questionnaire—physical, psychological, social relationships and environment—administered to the G1 (intervention group, *N* = 40) before and after treatment with *Monarda didyma* L. In **A**, the mean score of the WHOQOL-BREF questionnaire is compared before (T0) and after (T1) treatment in the G1 group. In **B**, the mean score of each domain of the questionnaire is compared, again analyzed in the G1 group before (T0) and after (T1) treatment. Data are presented as mean ± SD. Student’s *T*-test was used for statistical analysis; *, *p* < 0.05; **, *p* < 0.01. **C**, **D**, Correlation curves between the Sleep Index and the difference in DNAmAge from follow up to baseline in G1 (**C**; intervention group, *N* = 40) and G2 (**D**; placebo group, *N* = 41). Simple linear regression plots show the correlation in G1 and G2 groups between the Sleep Index and DNAmAge T1-T0 in G1 (**C**, *r* =  − 0.438806 and *p* = 0.0084) and G2 (**D**, *r* =  − 0.124919 and *p* = 0.4549) groups. Mean, standard error (SE), and 95% coefficient intervals (CI) are represented as green, pink, and black lines, respectively

The G1 group showed an increase in the quality of life score (WHOQOL-BREF) after supplementation with *Monarda didyma* L. extract (Fig. 6A, p = 0.0085), particularly in the physical domain (Fig. 6B, p = 0.0348). On the other hand, no significant changes were found in the scores of the other questionnaires on work ability (WAI), sleep quality and global assessment, neither between T0 and T1 nor between the G1 and G2 groups (Supplementary Table 4, *p* > 0.05). Similarly, there were no significant differences between G1 and G2 groups in physical parameters (steps, distance, running distance, calories) and sleep parameters (total sleep time, deep sleep time, light sleep time, REM time) measured by MiBand 7 throughout the study (Supplementary Table 5, *p* > 0.05).

Furthermore, there was variability in the total minutes of sleep per night between data collected by the MiBand 7 wearable device and self-reported responses to the PSQI questionnaire, with participants reporting less sleep (mean 47.44 min) than the device data indicates (Supplementary Fig. 3).

#### Effect size of biological aging parameters and quality of life

As shown in Table 3, the effect size trends were consistently positive for the intervention group (G1) and predominantly negative for the placebo group (G2), except for DNAmAge which showed a positive trend also for the G2 group. A binomial test yielded a statistically significant result (*p* < 0.004), confirming that the systematic trend favoring G1 was not due to chance.

**Table 3:** Effect size of biological aging parameters and quality of life

Endpoints	Effect size (G1)	Effect size (G2)
LTL (T/S)	0.3155	− 0.5858
DNAmAge (years)	0.1201	0.7630
Biological Age (K-D method)	0.1085	− 0.0950
WHOQOL-BREF: physical domain	0.3458	− 0.1276
WHOQOL-BREF: overall mean	0.4382	− 0.1605

#### Movement and Sleep Indexes (MI and SI) derived by MiBand 7 data

The G1 group had a significantly higher number of subjects with increased MI and SI than the G2 group (Table 4, MI *p* = 0.0475 and SI *p* = 0.0557). On the other hand, the number of subjects with stable and decreased MI and SI during the trial did not differ significantly between the two groups (G1 and G2) (Table 4, *p* > 0.05).

**Table 4:** Movement and Sleep Indexes derived by MiB and 7 data from *n* = 73 study participants

	*G1*		*G2*		Mann–Whitney *U* test *p*	
	Mean ± SD	*N* subject (%)	Mean ± SD	*N* subject (%)		Chi-square *p*
***Movement Index (0–1)***	0.50 ± 0.15	35(100%)	0.50 ± 0.14	38 (100%)	0.9694	0.6195
Increased	0.47 ± 0.18	16 (45.7%)	0.46 ± 0.15	9 (23.7%)	0.9779	**0.0475**
Stable	0.55 ± 0.13	14 (40.0%)	0.52 ± 0.14	20 (52.6%)	0.7692	0.2798
Decreased	0.45 ± 0.10	5 (14.3%)	0.49 ± 0.10	9 (23.7%)	0.5185	0.3082
***Sleep Index (0–1)***	0.41 ± 0.06	35(100%)	0.40 ± 0.07	38 (100%)	0.4976	0.2994
Increased	0.44 ± 0.05	7 (20.0%)	0.39 ± 0.10	2 (5.26%)	0.6667	0.0557
Stable	0.38 ± 0.06	10 (28.6%)	0.40 ± 0.05	18 (47.4%)	0.8600	0.0989
Decreased	0.42 ± 0.05	18 (51.4%)	0.40 ± 0.08	18 (47.4%)	0.8147	0.7289

Bold character is displayed only for significant values (*p*<0.05); Mann-Whitney *U* test is performed for unpaired continuous variables; Chi-square test is performed for categorical variables

#### Correlation between Movement or Sleep Indexes and the difference in biological aging indicators

Simple linear regression analyses (Supplementary Fig. 4 A–F) revealed that there is no significant correlation between the MI and the differences in biological aging indicators between T1 and T0 (i.e., DNAmAge T1-T0, LTL T1-T0, hematological ageT1-T0) in both groups G1 (Supplementary Fig. 4 A–C, *p* > 0.05) and G2 (Supplementary Fig. 4 D–F, *p* > 0.05).

However, a significantly positive correlation was observed between the SI and the difference in DNAmAge at T1 and T0 (DNAmAge T1-T0) in the G1 group (Fig. 6C, p = 0.0084), but not in the G2 group (Fig. 6D, p > 0.05). Additionally, there were no significant correlations between the SI and all other differences in biological aging indicators (i.e., LTL T1-T0 and hematological ageT1-T0) in both groups, G1 (Supplementary Fig. 5 A–B, *p* > 0.05), and G2 group (Supplementary Fig. 5 C–D, *p* > 0.05).

## Discussion

The growing aging population in developed countries has led to increased societal and economic burdens [53] (https://www.who.int/news-room/fact-sheets/detail/ageing-and-health), highlighting the urgent need for therapies that support healthy aging. One promising research area focuses on botanical extracts which are rich in phytoconstituents and have the potential to be developed into agents that enhance healthspan. Compounds derived from natural sources that target key aspects of cellular aging have been shown to improve both healthspan and lifespan in various models, including cell cultures [54], invertebrates, rodents, and humans [27]. These phytochemicals act as geroprotective agents by reducing oxidative stress, promoting cellular repair, and eliminating senescent cells in tissues, improving age-related physiological phenotypes, and acting as “senomorphics” to inhibit inflammation and immune senescence [27]. Consequently, botanical extracts are a valuable area of study for developing treatments that can help mitigate the societal and economic impacts of an aging population [54]. Reducing oxidative stress—a central factor in cellular aging and degenerative diseases—preventing telomere damage and modulating DNA methylation have been demonstrated to create a healthier tissue environment [55–57]. This, in turn, decreases low-grade inflammation, which drives age-related diseases [58]. The observed geroprotective effects of *Monarda didyma* L. extract in both in vitro studies and the clinical trial, particularly its role in mitigating oxidative stress and inflammation—likely mediated by its principal flavonoid DID [28, 32, 33, 59]—highlight its potential as an anti-aging dietary supplement to promote health in aging populations. The prominent presence of DID establishes it as the primary candidate responsible for the extract’s observed geroprotective effects.

### In vitro studies on *Monarda didyma* L. extract

In the present study, we describe a natural extract that displays in vitro antioxidant properties, slows the rate of telomere shortening, and protects against DNA damage with reduction of cellular senescence and improvement of endothelial function and vascular permeability.

#### Antioxidant properties

The *Monarda didyma* L. extract’s strong antioxidant properties were evident, as demonstrated by its capacity to reduce protein carbonylation levels. This antioxidant activity is essential in combating oxidative stress, a major contributor to cellular aging and various degenerative diseases [7]. The ability to reduce oxidative damage further supports the extract’s role in promoting cellular health and longevity.

#### Slowing telomere shortening rate

Cells treated with the *Monarda didyma* L. extract for 6 weeks showed a significant reduction in the rate of telomere shortening compared to untreated control cells. It can be hypothesized that the molecules found in *Monarda didyma* L. extract play a significant role in safeguarding DNA from damage. Certain polyphenols are renowned for their ability to stabilize and protect the DNA structure, including telomeres. By shielding DNA from harm, these molecules may help mitigate the wear and breakage of telomeres during cellular replication [60]. Moreover, *Monarda didyma* L. extracts exhibit potent antioxidant properties, capable of reducing oxidative stress within cells [54]. Oxidative stress is a major contributor to telomere shortening, as free radicals can inflict damage on DNA, telomeres included [61]. Furthermore, the activation of telomerase by *Monarda didyma* L. as a possible mechanism remains unverified at this time.

The biological significance of the finding that *Monarda didyma* L. extract can slow telomere shortening is important for understanding aging and cellular health. Telomeres are protective caps at the ends of chromosomes that shorten with each cell division, leading to cellular aging and senescence when they become too short [45]. By slowing telomere shortening, *Monarda didyma* L. extract may extend the lifespan of cells, helping to maintain tissue function and delay age-related decline. This has several key implications including extending cellular lifespan as cells can divide more times before reaching senescence, thus maintaining tissue health longer; delaying age-related diseases as slower telomere shortening can delay the onset of diseases linked to aging, such as cardiovascular diseases, neurodegenerative disorders, and certain cancers [12]. Improving regenerative capacity as tissues that regenerate frequently, like skin and blood, can benefit from enhanced repair and regeneration. *Monarda didyma* L. extract shows significant promise as a gerotherapeutic agent by slowing telomere shortening and targeting cellular aging processes. Developing it into a therapy could promote longevity and enhance quality of life, highlighting its broad implications for health and well-being.

#### Protection against DNA damage

The *Monarda didyma* L. extract induces a reduction in γH2A.X and H3K9me3 levels in genotoxically stressed cells. γH2A.X is a marker of DNA double-strand breaks [62], while H3K9me3 is associated with chromatin changes following DNA damage [63]. Lower levels of these markers indicate reduced DNA damage and improved DNA repair mechanisms [63, 64].

By protecting DNA from damage, the extract plays a crucial role in maintaining genomic stability. Genomic instability is an early hallmark of cellular aging [2]. It can lead to mutations, loss of genetic information, and chromosomal abnormalities, all of which are linked to cancer and other diseases. DNA damage also contributes to cellular senescence and aging [12]. Therefore, by reducing DNA damage, the extract may help prevent premature aging, promoting healthier cell function and longevity.

This protective effect underscores the extract’s potential as a valuable agent in safeguarding genomic integrity. The significant reduction in DNA damage markers highlights the extract’s role in maintaining genomic stability and mitigating the adverse effects of cellular aging.

#### Reduction in cellular senescence

The extract significantly reduced cellular senescence, as indicated by decreased SA-β-galactosidase activity in aged cells. SA-β-galactosidase (senescence-associated β-galactosidase) activity is a widely used biomarker to identify senescent cells [65]. High levels of SA-β-galactosidase activity are typically present in aged or senescent cells [66]. Therefore, a decrease in SA-β-galactosidase activity suggests a reduction in the number of senescent cells. By reducing cellular senescence, the *Monarda didyma* L. extract may help maintain tissue function and delay the onset of age-related dysfunctions. This could lead to improvements in healthspan and potentially lifespan, as well as provide therapeutic benefits for diseases where senescence plays a critical role. This reduction suggests a geroprotective effect at the cellular level, potentially reversing age-related cellular markers.

#### Improved endothelial function and reduced vascular permeability

Additionally, *Monarda didyma* L. extract improved endothelial function and reduced vascular permeability, indicating potential cardiovascular benefits. The reduction in pro-inflammatory markers, such as VCAM and intracellular ROS levels, highlights the extract's anti-inflammatory and antioxidant effects, which are critical for maintaining vascular health and preventing cardiovascular diseases [67].

While additional studies are needed to ensure this treatment does not impact other mechanisms linked to extending healthspan, the findings show a notable reduction in cellular senescence, preservation of telomere length, decreased DNA damage, and strengthened antioxidant defenses. Together, these effects position the extract as a promising candidate for geroprotective therapies and as a promising dietary supplement for aging populations.

### Clinical trial

One of the key strengths of our study is the focus on the effects of *Monarda didyma* L. extract in human treatment. Our clinical trial offers compelling evidence of the extract’s geroprotective potential. The study’s randomized, double-blind design, coupled with age and sex matching between the intervention group (G1) and the control group (G2), strengthens the reliability of our findings by minimizing bias.

#### Biological aging

Another strength of our study is the use of multiple methods to assess biological aging. We evaluated both mitotic and non-mitotic pathways using LTL, DNAmAge, and the hematological age according to the method of Klemera and Doubal [51]. This comprehensive approach provides a thorough and accurate assessment of the aging process, further enhancing the validity of our findings.

The study revealed notable differences in biological age markers between the intervention group (G1) and the control group (G2). The LTL, a key marker of cellular aging, showed an increase in the G1 group, indicating a potential protective effect of *Monarda didyma* L. extract on telomere integrity. Conversely, the G2 group experienced a significant decrease in LTL, suggesting a lack of protective effect and possibly an acceleration of cellular aging processes in the absence of the extract intake. Furthermore, the LTL post-treatment was significantly longer in G1 compared to G2, underscoring the extract’s potential role in maintaining telomere length and promoting cellular longevity.

LTL results observed in both in vitro and in clinical settings, also suggest a potential role of *Monarda didyma* L. extract in delaying endothelial cell senescence and maintaining vascular integrity, as telomere shortening is associated with vascular dysfunction, atherosclerosis, and increased cardiovascular risk [68]. These implications for cardiovascular health were further supported by the effects that the extract exerts in vitro on endothelial function through a dual mechanism involving the reduction of ROS and suppression of VCAM expression, which in turn reduces vascular inflammation and its key role in the pathogenesis of atherosclerosis and other cardiovascular diseases [69]. These mechanisms, together with its anti-inflammatory and antioxidant properties, position *Monarda didyma* L. extract as a promising candidate for geroprotective therapies and dietary interventions aimed at promoting cardiovascular health in aging populations.

While our findings on telomere length are significant, we acknowledge that these changes might be transient and potentially reflect immune activation. This is supported by studies showing increased telomere length in younger T cells during inflammatory responses [70, 71]. However, unlike our previous research, where increased telomere length in smokers was attributed to the recruitment of younger inflammatory cells triggered by smoke-induced inflammatory signals [48], this study excluded smokers as part of the eligibility criteria. Additionally, in G1 group, no changes in inflammation biomarkers or leukocyte populations were observed at the end of the supplementation period compared to G2 group. This further reduces the likelihood that the observed telomere length changes were driven by immune activation, providing a stronger basis for attributing these effects to the supplementation itself.

In addition to LTL, epigenetic aging, assessed through DNAmAge, displayed divergent trends between the groups. DNAmAge remained stable in the G1 group, indicating a mitigation of epigenetic aging. However, the G2 group exhibited a significant increase in DNAmAge, pointing to a greater rate of epigenetic aging in the absence of *Monarda didyma* L. extract. This divergence suggests that the extract may have a stabilizing effect on the epigenetic clock, potentially slowing the biological aging process. Indeed, the stabilization observed in G1 may reflect the geroprotective properties of the supplement, consistent with a previous study demonstrating its impact on molecular pathways associated with aging [34]. These findings align with the hypothesized mechanism of action. Furthermore, to ensure that the observed differences between G1 and G2 are not attributable to random variability, we analyzed variability within and between groups, providing confidence intervals to support the robustness of our findings. In addition, we have rigorously detailed our randomization, blinding, and compliance monitoring processes and confirmed the consistency of sample handling and analysis protocols to avoid the influence of factors such as baseline characteristics, compliance, or measurement biases on the observed divergences. Although the randomized, double-blind, age- and sex-matched design reduces the impact of random variation, inherent biological variability—especially in smaller cohorts—cannot be entirely excluded. Larger, more diverse studies will help confirm these findings and ensure robustness. Potential confounders such as lifestyle or dietary differences could influence outcomes. However, the randomized design minimizes these risks. Future studies should incorporate stricter controls to further enhance reproducibility and reliability.

These observations on biological aging markers in humans are consistent with our in vitro findings, where *Monarda didyma* L. has been shown to impact telomere length positively without altering epigenetic age. This alignment between human and in vitro results underscores the potential of extract as a therapeutic natural agent for preserving telomere length and reducing the pace of epigenetic aging.

In our study on the effects of *Monarda didyma* L. extract, we did not observe significant changes in hematological age between groups post-treatment. Hematological age was calculated using the Klemera and Doubal [51], chosen for its standardization and widespread acceptance in the calculation of biological age, based on the data from our study. However, we acknowledge that its use presents certain limitations and interpretative challenges due to its exploratory nature. The lack of observed variation may be attributed to the specific parameters included in the calculation, which might not capture the hematological markers affected by our intervention. This underscores the need for further refinement and validation of this tool to enhance its applicability and reliability in aging research. Furthermore, while *Monarda didyma* L. extract demonstrated beneficial effects on biological aging markers such as LTL and DNAmAge, these markers were not included in the hematological age calculation. This highlights the necessity of developing more advanced algorithms that integrate a broader spectrum of biological and clinical markers to better capture the full impact of geroprotective treatments.

##### Biological significance of DNA methylation changes

Significant changes in DNA methylation patterns were observed, with the control group (G2) showing hypermethylation at specific sites such as ELOVL2 and FHL2 compared to the intervention group (G1). ELOVL2 (Elongation of Very Long Chain Fatty Acids Protein 2) is a well-established biomarker for aging [72]. Hypermethylation at this site is commonly associated with increased biological age and occurs in several tissues [73, 74]. The hypermethylation observed in the control group suggests an accelerated aging process compared to the intervention group. FHL2 (Four and a Half LIM Domains 2) functions as an interaction platform, regulating protein signalling pathways through various protein–protein interactions [75–77]. While FHL2 is well-studied in oncology, cardiovascular diseases, inflammation, and cell differentiation, its role in metabolism has only recently gained attention [78]. Interesting hypermethylation of FHL2 correlates with islet expression of multiple genes, including FHL2. Silencing these genes in β-cells alter insulin secretion and associate with insulin secretion in vivo and T2D [78]. The hypermethylation of FHL2 in the placebo group suggests potential disruptions in these critical cellular functions. These findings imply that *Monarda didyma* L. extract may influence methylation processes, thereby contributing to the modulation of aging at the epigenetic level. This further implies that the extract may help maintain normal cellular function by preventing adverse epigenetic modifications.

The active compounds in *Monarda didyma L.* extract likely exhibit effects similar to well-studied dietary molecules such as epigallocatechin gallate, quercetin, sulforaphane, curcumin, genistein, resveratrol, apigenin, and gallic acid, which are known to inhibit DNA methyltransferases (DNMTs) [79]. These compounds selectively reduce DNA methylation in critical regions, reactivating genes involved in cellular repair and longevity. The potential role of DID, a key component of *Monarda didyma* L*.*, in DNMT inhibition merits further exploration, particularly given the synergistic effects observed among polyphenols in enhancing epigenetic modulation.

#### Comprehensive evaluation of lifestyle parameters and quality of life

A key strength of our study is the thorough evaluation of essential lifestyle factors, such as sleep and physical activity, which are closely linked to healthy aging. We assessed these factors using both subjective questionnaires and objective measurements from the MiBand 7 wearable device. This dual approach provided a more comprehensive and accurate understanding of the improvements resulting from *Monarda didyma* L. extract treatment.

The intervention group (G1) reported significant improvements in questionnaire data on quality of life, especially in the physical domain, compared to the control group (G2). The significant improvement in the physical domain of quality of life among G1 participants suggests that *Monarda didyma* L. extract may support better physical function. This finding aligns with the observed increase in the computed MI, indicating more frequent or vigorous physical activity, during the study, which is essential for healthy aging and the prevention of chronic diseases. Improvements in overall well-being suggest that the extract positively impacts both mental and physical health. The subjective feelings of enhanced well-being could be linked to reduced stress, better sleep quality, and improved physical health, all contributing to a higher quality of life [80–82]. The biological plausibility of these findings is supported by several mechanisms through which *Monarda didyma* L. extract may exert its beneficial effects. In addition to the well-known antioxidant effects of DID [28], which reduce oxidative stress and protect cells from damage, its anti-inflammatory properties lead to improved physical function and reduced pain, thereby enhancing the receipted quality of life. Research has shown that DID, a leading constituent of *Clinopodium mexicanum* and widely present in *Monarda fistulosa* L*.* other than in *Monarda didyma* L. two of the most popular species of the plant-, possesses significant anxiolytic-like and antinociceptive properties [83]. These effects are mediated through the GABAergic system, which is crucial for regulating mood, anxiety, and pain perception [84]. By reducing anxiety and alleviating pain, the anxiolytic-like effects of DID can contribute to increase physical activity and improve sleep quality, mental health, and the overall quality of life.

#### Movement and sleep indices improvements

Our research observed a greater number of subjects with an increase in both MI and SI during the study in the G1 group than in the G2 group, providing further evidence of improvement in the physical domain and sleep quality. Our results suggest that interventions initially aimed at improving molecular targets, such as LTL and DNAmAge, also had positive phenotypic effects, including increased physical activity and better sleep quality, thereby enhancing overall health and well-being in accordance with the observed improvements in quality of life (WHOQOL-BREF). These findings align with current evidence [17, 18, 85–88] highlighting the role of non-pharmacological interventions in promoting longevity by targeting oxidative stress and chronic inflammation, key drivers of cellular aging.

Furthermore, the increase in physical activity, as measured by the MI, is known to improve cardiovascular health, regulate metabolism, and strengthen the musculoskeletal system [89]. Similarly, the improvement in sleep quality, indicated by the increase in the SI, supports important cognitive functions such as memory and problem-solving [90], strengthens the immune system [91], regulates hormones [92], and facilitates cellular repair [93] and detoxification processes [94]. These processes are essential for maintaining neuronal health and preventing neurodegenerative diseases.

Furthermore, the study highlights the importance of using both subjective and objective measures to assess the effects of interventions on sleep. This comprehensive approach is essential to gain a full understanding of the extract's impact on sleep and overall health.

#### Correlation between sleep index and epigenetic aging

Our study revealed a positive correlation between improved sleep and reduced epigenetic aging in the treatment group (G1). This indicates that DID may directly influence DNA methylation patterns while also reducing oxidative stress and chronic inflammation, leading to a younger epigenetic profile. Melatonin, widely recognized as the master regulator of circadian rhythm, also plays a role in genomic stability and epigenetic modifications, including DNA methylation [95]. Its antioxidative properties help reduce ROS and reactive nitrogen species (RNS), activating antioxidant enzymes. Additionally, melatonin has been shown to influence DNA methylation, with variations observed in night shift workers and during embryonic development [95]. These findings emphasize the dual benefits of DID, enhancing both molecular markers and physiological health, particularly sleep quality. This underscores the holistic impact of DID, suggesting that it not only operates through molecular mechanisms but also improves vital physiological functions like sleep, promoting overall health and longevity.

#### Basic biochemical parameters

Another strength of our study is evaluating how classical commonly used basic biochemical parameters were affected by *Monarda didyma* L*.* extract.

##### Hematological and metabolic changes

The placebo group (G2) showed unfavorable changes in hematocrit, neutrophils, monocytes, MCV, RDW and IL-6 levels, potentially reflecting an inflammatory or stress response. In contrast, the intervention group (G1) did not show these alterations, suggesting a possible protective or stabilizing effect of *Monarda didyma* L. extract on these parameters. Stable creatinine and eGFR levels in the G1 group suggest maintenance of good renal function, while we observed a slight decrease in creatinine levels and a slight increase in eGFR levels, still within normal reference values [96], in participants of the G2 group after treatment. The calculation of eGFR is based on the concentration of creatinine in the blood [97]. Creatinine, produced by muscles and eliminated by the kidneys, is reduced in the blood when kidney function is efficient [96].

##### Lipid profile

Additionally, the G2 group exhibited elevated levels of HDL and reduced levels total cholesterol/HDL and LDL/HDL cholesterol compared to the G1 group. However, it should be noted that these differences in lipid profile could be attributed to their values at baseline, with the G2 group having higher values at baseline, before treatment.

##### Blood glucose and cortisol levels

Both groups showed reductions in glycemia and salivary cortisol post treatment, but these changes were more pronounced in G1, indicating that the extract may help regulate blood glucose and stress levels. These results suggest a general improvement in stress and glucose metabolism.

In summary, these biochemical results highlight the potential beneficial effects of *Monarda didyma* L. extract on various health markers. The lack of significant adverse changes in the intervention group, in contrast to several deteriorations in the control group, suggests a protective role of the extract.

The primary biological mechanism by which *Monarda didyma* L. extract exerts its effects appears to be through its anti-inflammatory and antioxidant properties, which in our powdered extract can be attributed to the presence of DID [28]. These properties help mitigate oxidative stress and inflammation, which are key contributors to cellular damage, kidney function deterioration, and metabolic imbalances. By reducing oxidative stress and inflammation, the extract helps maintain genomic stability, supports renal function, and promotes healthier metabolic profiles.

These biochemical results, consistent with our in vitro results, highlight the potential beneficial effects of *Monarda didyma* L. extract on various health markers. Future studies should explore these biochemical changes in more depth, considering baseline differences and controlling for potential confounding variables to fully understand the extract's impact on metabolic and cardiovascular health.

### Limitations and strengths of the study

#### Limitations

While our study provides important insights into the potential geroprotective effects of Monarda didyma L. extract, certain limitations must be acknowledged. The relatively small sample size and short study duration, although statistically justified, restrict the ability to draw conclusions about long-term effects or broader population applicability. However, participants’ affiliation with the University of Padua provides an opportunity for follow-up studies to evaluate sustained outcomes. Baseline differences between participants, along with the subjective nature of self-reported measures such as quality of life and physical activity, introduce potential biases. Although our randomized, double-blind, age-, and sex-matched design minimizes these risks, future studies could benefit from stricter controls on diet, lifestyle, and environmental factors to further enhance data reliability. Additionally, while the observed stabilization of epigenetic age and telomere length suggests geroprotective potential, these findings require cautious interpretation. The effects, although promising, necessitate further exploration through mechanistic studies and validation in larger, diverse cohorts to confirm their broader applicability and long-term benefits.

#### Strengths

Despite these limitations, the study has significant strengths. The in vitro component was designed with meticulous attention to detail, including preliminary dose–response experiments to determine effective concentrations of *Monarda didyma* L. extract for specific cell types and experimental conditions. This systematic approach ensures that the findings are biologically meaningful and robust. The selection of diverse cell lines, tailored to model specific aging-related processes such as oxidative stress and cellular senescence, enhances the translational relevance of the preclinical results. Furthermore, the clinical trial’s randomized, double-blind design and age- and sex-matched groups strengthen the validity of the findings and reduce the likelihood of confounding effects. The inclusion of calculated effect sizes is a key strength, enhancing both the validity and interpretability of the study’s findings. The consistent positive trends in the intervention group (G1), contrasted with the predominantly negative trends in the placebo group (G2), and supported by robust statistical analysis (*p* < 0.004), strongly highlight the efficacy of *Monarda didyma* L. extract. However, it would be valuable to further emphasize how these preliminary observations provide a solid foundation for future, larger-scale studies. Analyzing rates of change and effect sizes in broader contexts could confirm the observed benefits and provide a more precise estimation of their clinical impact. Rigorous statistical adjustments were implemented to minimize the risk of type I errors; however, results near the significance threshold should be interpreted with caution. Despite this, the consistent evidence from various biomarkers and endpoints highlights the potential geroprotective benefits of *Monarda didyma* L. extract. The integration of biological markers, such as LTL and DNAmAge, alongside assessments of quality of life, physical activity, and biochemical parameters, provides a comprehensive and multidimensional evaluation. This approach bridges preclinical and clinical findings, establishing a robust foundation for future research on the role of *Monarda didyma* L. extract in promoting healthy aging.

### Comparative potential of *Monarda didyma* L. in oxidative stress, telomere preservation, and DNA methylation

Our findings suggest that *Monarda didyma* L. extract has notable potential in mitigating oxidative stress, telomere shortening, and DNA methylation changes. While direct comparisons with other botanicals or therapies were not part of this study, existing evidence suggests that *Monarda didyma* L. shows antioxidant effects comparable to compounds like resveratrol in preliminary models; unlike telomerase activators (e.g., TA-65), *Monarda didyma* L. indirectly preserves telomere length by reducing oxidative damage; the extract may modulate methylation patterns similarly to sulforaphane-rich broccoli sprout extracts.

Furthermore, previous research on *Monarda didyma* L., particularly its essential oil, demonstrates its capacity to improve learning and memory by modulating Nrf2 and MAPK pathways, suggesting potential neuroprotective benefits [34]. These results are in line with existing evidence for neuroprotective effects of other natural compounds and botanicals via antioxidant and anti-inflammatory mechanisms, such as *Mucuna pruriens* [98], ursolic acid [99], and *Withania somnifera* [100] for Parkinson’s disease, and indole-derived compounds [101] and traditional medicinal plants [102] for Alzheimer’s diseases, emphasizing the potential of natural products to reduce oxidative stress and promote neuronal health.

## Conclusion

*Monarda didyma* L. extract demonstrates in vitro geroprotective potential through various mechanisms, including reducing cellular senescence, protecting telomere length, mitigating DNA damage, and enhancing antioxidant defenses. The clinical trial findings support these benefits, showing improvements in biological aging markers, quality of life and physical activity, and biochemical parameters. These results suggest that *Monarda didyma* L. extract may be a valuable component in anti-aging therapies and dietary supplements to promote health and longevity in aging populations. Notably, the inclusion of *Monarda didyma* L. in Italy’s regulatory framework for botanicals underscores its safety and suitability for human consumption, supporting its potential use in personalized dietary interventions. However, further research is needed to fully understand the effects of this extract and to explore its potential applications in clinical settings. This study underscores the potential of safe, effective, and sustainable botanical extracts in the development of therapies that can mitigate the societal and economic impact of an aging population.

## Supplementary Information

Below is the link to the electronic supplementary material.

Supplementary file1 (DOC 664 KB)

## Data Availability

The datasets generated and analyzed during the current study are available from the corresponding author upon reasonable request.

## Declarations

The clinical trial was conducted in accordance with the Declaration of Helsinki and approved by the Ethics Committee of the University of Padova (Approval No. 5518/AO/22; Study Code: EMODISU) on December 6, 2022. All participants provided written informed consent prior to enrollment.
