# Supplementary material for: Unveiling the geroprotective potential of Monarda didyma L.: insights from in vitro studies and a randomized clinical trial on slowing biological aging and improving quality of life
Source: GeroScience. 2025 Mar 10;47(3):4253–90. doi: 10.1007/s11357-025-01580-2 (PMC12181487; doi:10.1007/s11357-025-01580-2)
Supplement: Supplementary file 1 — Supplementary file1 (DOC 664 KB) [file 11357_2025_1580_MOESM1_ESM.doc]

**Supplementary Material for:**

**Unveiling the geroprotective potential of *Monarda didyma* L.: insights from in vitro studies and a randomised clinical trial on slowing biological aging and improving quality of life**

Manuela Campisi^1†^, Luana Cannella^1†^, Omar Paccagnella^2^, Alessandra R. Brazzale^2^, Alberto Agnolin^2^, Torsten Grothe^3^, Julia Baumann^3^, Sofia Pavanello^1,4,5*^

^1^ Department of Cardiac, Thoracic, and Vascular Sciences and Public Health, University of Padua, Padua, Italy.

^2^ Department of Statistical Sciences, University of Padua, Padua, Italy.

^3^ Mibelle Group Biochemistry, Bolimattstrasse 1, 5033 Buchs, Switzerland.

^4^ University Hospital of Padova, Occupational Medicine Unit, Padua, Italy.

^5^ Centre of Studies and Activities for Space CISAS "G. Colombo" of University of Padua, Padua, Italy.

^†^ Contributed equally

ORCID:

Manuela Campisi <https://orcid.org/0000-0002-7372-4136>

Luana Cannella <https://orcid.org/0000-0002-3827-3632>

Sofia Pavanello <https://orcid.org/0000-0002-5229-9900>

* Corresponding author

e-mail: [sofia.pavanello@unipd.it](mailto:sofia.pavanello@unipd.it)

**CONSORT Flow Diagram of enrollment, allocation, follow-up and analysis of participants in G1 and G2 groups in the clinical trial.**

***Enrollment***

Assessed for eligibility (n=904)

Excluded (n=723)

- Not meeting inclusion criteria (n=611)
- Declined to participate (n= 36)
- Other reasons (n=76)

Randomized (n= 81)

***Allocation***

Allocated to intervention G1 group (n=40)

- Received allocated intervention (n=40)
- Did not receive allocated intervention (n=0)

Allocated to intervention G2 group (n=41)

- Received allocated intervention (n=41)
- Did not receive allocated intervention (n=0)

***Follow-Up***

Lost to follow-up (n=0)

Discontinued intervention (n=0)

Lost to follow-up (n=0)

Discontinued intervention (n=0)

***Analysis***

Analysed (n=40)

- Excluded from analysis (n=0)

Analysed (n=41)

- Excluded from analysis (n=0)

**CONSORT 2010 Flow Diagram**. The flow diagram illustrates the enrollment, allocation, follow-up and analysis of participants in the clinical trial. A total of 904 individuals were screened during routine health assessments at the Occupational Medicine Unit – AOUP, of whom 723 were excluded (611 did not meet the inclusion criteria, 36 declined to participate, and 76 for other reasons). A total of 81 participants were randomized into two groups: n=40 in G1 group and n=41 in G2 group. All participants completed the allocated intervention, with no losses to follow-up or discontinuations. The data from all participants were analyzed, with no exclusions, ensuring the integrity of the study results. This transparent reporting adheres to CONSORT guidelines, providing a clear overview of participant progress throughout the clinical trial.

**Supplementary Tables**

**Supplementary Table 1. Study population (N=81) characteristics at Enrollment visit (T0).**

| ***Variable*** | ***Mean±SD*** | ***N subjects*** | ***%*** |
| --- | --- | --- | --- |
| *Age (years)* | 55.72±4.64 |  |  |
| *Gender:* |  |  |  |
| *M* |  | 40 | 49.38 |
| *F* |  | 41 | 50.62 |
| *Marital status:* |  |  |  |
| *not married* |  | 11 | 13.58 |
| *married* |  | 50 | 61.73 |
| *cohabiting* |  | 13 | 16.05 |
| *divorced* |  | 6 | 7.41 |
| *widower* |  | 1 | 1.23 |
| *Years of education (years):* | 18.49±4.17 |  |  |
| *Lower (high school diploma)* | 12.35±1.42 | 20 | 24.69 |
| *Medium (Bachelor’s degree, Master’s degree)* | 17.53±1.12 | 19 | 23.46 |
| *High (Postgraduation degree, PhD)* | 21.86±1.42 | 42 | 51.85 |
| *Weight (kg)* | 71.37±12.98 |  |  |
| *Height (m)* | 1.70±0.08 |  |  |
| *Abdominal circumference (cm)* | 88.88±11.82 |  |  |
| *BMI (Kg/m^2^):* | 24.54**±**3.18 |  |  |
| *Underweight (*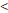*18.5)* |  | 2 | 2.47 |
| *Normal range (*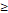*18.5;<25.0)* |  | 44 | 54.32 |
| *Overweight (*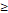*25.0; <30.0)* |  | 32 | 39.51 |
| *Obese (≥30.0)* |  | 3 | 3.70 |
| *Systolic blood pressure (mmHg)* | 123.64±16.10 |  |  |
| *Diastolic blood pressure (mmHg)* | 81.06±9.35 |  |  |
| ***EMPLOYMENT ANAMNESIS*** |  |  |  |
| *Professional position:* |  |  |  |
| *professor/researcher/doctor* |  | 33 | 40.74 |
| *laboratory or informatic technician* |  | 22 | 27.16 |
| *administrative technician or librarian* |  | 26 | 32.10 |
| *Years of work in the current job (years)* | 17.49±9.96 |  |  |
| *Performance of night shifts* |  | 2 | 2.47 |
| *Work risks:* |  |  |  |
| *none* |  | 4 | 4.94 |
| *chemical* |  | 34 | 41.98 |
| *biological* |  | 31 | 38.27 |
| *VDT* |  | 34 | 41.98 |
| *other* |  | 3 | 3.70 |
| ***LIFESTYLE*** |  |  |  |
| *Tobacco habit:* |  |  |  |
| *Ex-smoker* |  | 21 | 25.93 |
| *Non-smoker* |  | 60 | 74.07 |
| *Exposure to second-hand smoke* |  | 5 | 6.17 |
| *Pack/years [(cigarettes/20) per years of smoking]* | 5.54±5.46 |  |  |
| *Alcohol consumption:* |  |  |  |
| *daily (0,5 or 1 UA)* |  | 34 | 41.98 |
| *occasionally* |  | 28 | 34.57 |
| *non-consumer* |  | 19 | 23.46 |
| *Physical activity* |  | 64 | 79.01 |
| *Type of physical activity:* |  |  |  |
| *none* |  | 17 | 20.99 |
| *walking* |  | 21 | 25.93 |
| *running* |  | 12 | 14.81 |
| *cycling* |  | 11 | 13.58 |
| *gym/yoga* |  | 27 | 33.33 |
| *other sports (padel, swimming, rowing, tennis, dancing, football, etc..)* |  | 13 | 16.05 |
| *Years of physical activity:* | 15.59±13.19 |  |  |
| *0-3 years* |  | 13 | 16.05 |
| *3-10 years* |  | 21 | 25.93 |
| *>10 years* |  | 30 | 37.04 |
| *Frequency of vegetable meals (n/week)* | 10.22±3.05 |  |  |
| *Frequency of fruit meals (n/week)* | 8.77±4.11 |  |  |
| *Indoor pollution - heating:* |  |  |  |
| *wood stove* |  | 6 | 7.41 |
| *pellet stove* |  | 3 | 3.70 |
| *alcohol/bioethanol stove* |  | 2 | 2.47 |
| *gas* |  | 79 | 97.53 |
| *photovoltaic panels* |  | 4 | 4.94 |
| *Fireplace in the house (wood and pellet)* |  | 11 | 13.58 |
| *Living area:* |  |  |  |
| *non-urban/rural area* |  | 16 | 19.75 |
| *urban/peripheral area* |  | 65 | 80.25 |
| *House close to industrial settlements* |  | 12 | 14.81 |
| *Traffic in the living area:* |  |  |  |
| *continuous intense for a good part of the day* |  | 24 | 29.63 |
| *intermittent intense* |  | 32 | 39.51 |
| *scarce or absent* |  | 25 | 30.86 |
| *Means of transport usually used:* |  |  |  |
| *on foot* |  | 29 | 35.80 |
| *bike* |  | 39 | 48.15 |
| *car and motorbike* |  | 49 | 60.49 |
| *bus/tram/train* |  | 11 | 13.58 |
| *Total travel time:* |  |  |  |
| *up to 30 min* |  | 15 | 18.52 |
| *30-60 min* |  | 34 | 41.98 |
| *> 60 min* |  | 32 | 39.51 |
| ***Covid-19 infection before the clinical trial****:* |  |  |  |
| *none* |  | 26 | 32.10 |
| *once* |  | 47 | 58.02 |
| *reinfection* |  | 8 | 9.88 |
| *Vaccine doses:* |  |  |  |
| *0* |  | 1 | 1.23 |
| *1* |  | 1 | 1.23 |
| *2* |  | 5 | 6.17 |
| *3* |  | 46 | 56.79 |
| *4* |  | 28 | 34.57 |

**Supplementary Table 2. Methylation levels (% met) of five selected markers before (T0) and after treatment (T1) in G1 and G2 groups.**

|  | **G1** | | | **G2** | | | **G1 vs G2**  **T0** | **G1 vs G2**  **T1** |
| --- | --- | --- | --- | --- | --- | --- | --- | --- |
| **Markers** | **T0 Methylation % (Mean±SD)** | **T1 Methylation % (Mean±SD)** | **p-value**  **(t-test)** | **T0 Methylation % (Mean±SD)** | **T1 Methylation % (Mean±SD)** | **p-value**  **(t-test)** | **p-value**  **(Mann-Whitney test)** | **p-value (Mann-Whitney test)** |
| ***ELOVL2*** | 70.62±5.54 | 70.61±5.24 | 0.9950 | 70.49±6.04 | 74.03±6.82 | **<0.0001** | 0.7189 | **0.0452** |
| ***C1orf132*** | 58.45±4.68 | 56.86±5.23 | **0.0001** | 55.98±7.13 | 54.97±6.78 | **0.0104** | 0.1477 | 0.2040 |
| ***TRIM59*** | 40.64±3.56 | 40.29±3.05 | 0.1457 | 41.16±3.80 | 41.01±3.94 | 0.6201 | 0.4692 | 0.2281 |
| ***KLF14*** | 9.238±1.67 | 9.100±1.80 | 0.3785 | 9.405±2.07 | 9.337±2.06 | 0.5747 | 0.8776 | 0.9416 |
| ***FHL2*** | 45.18±4.64 | 45.07±4.04 | 0.7015 | 47.02±4.02 | 46.92±4.11 | 0.6878 | 0.0676 | **0.0452** |

Bold character is displayed only for significant values (p<0.05);

T-test is performed for paired continuous variables; Mann-Whitney U test is performed for unpaired continuous variables.

**Supplementary Table 3. Hematological age measured before and after treatment in G1 (N=40) and G2 (N=41).**

| ***HEMATOLOGICAL AGE***  ***(YEARS)*** | ***G1*** | | | ***G2*** | | | ***G1 vs G2***  ***(T0)*** | ***G1 vs G2***  ***(T1)***  ***Mann-Whitney U test*** |
| --- | --- | --- | --- | --- | --- | --- | --- | --- |
|  | **T0**  **Mean±SD** | **T1**  **Mean±SD** | **Paired**  **t test** | **T0**  **Mean±SD** | **T1**  **Mean±SD** | **Paired**  **t test** | ***Mann-Whitney U test*** |  |
|  | 55.83±8.55 | 56.33±9.37 | 0.4965 | 55.61±8.92 | 55.12±8.99 | 0.5464 | 0.8454 | 0.3677 |

T-test is performed for paired continuous variables; Mann-Whitney U test is performed for unpaired continuous variables.

**Supplementary Table 4. Questionnaires about working capacity, quality of life, quality of sleep, global assessment test, group G1 (N=40) and group G2 (N=41).**

| ***QUESTIONARIES*** | ***G1*** | | | | | | | ***G2*** | | | | | | |
| --- | --- | --- | --- | --- | --- | --- | --- | --- | --- | --- | --- | --- | --- | --- |
|  | **T0**  **Mean±SD** | **T0**  **N subjects** | **T0**  **% subjects** | **T1**  **Mean±SD** | **T1**  **N subjects** | **T1**  **% subjects** | **Pearson χ^2^ Test^#^; Paired T-test^*^** | **T0**  **Mean±SD** | **T0**  **N subjects** | **T0**  **% subjects** | **T1**  **Mean±SD** | **T1**  **N subjects** | **T1**  **% subjects** | **Pearson χ^2^ Test**^#^**; Paired T-test^*^** |
| ***Work ability index*** *(WAI) [n (%)]:* |  |  |  |  |  |  | 0.6491 |  |  |  |  |  |  | 0.158 |
| *poor* |  | 0 | 0 |  | 0 | 0 |  |  | 0 | **0** |  | 0 | **0** |  |
| *moderate* |  | 3 | 7.50 |  | 2 | 5.00 |  |  | **0** | **0** |  | 0 | 0 |  |
| *good* |  | 20 | 50.00 |  | 17 | 42.50 |  |  | 17 | 41.46 |  | 10 | 24.39 |  |
| *excellent* |  | 17 | 42.50 |  | 21 | 52.50 |  |  | 24 | 58.54 |  | 31 | 75.61 |  |
| ***Quality of life****: World Health Organization’s Quality of Life Assessment (WHOQOL-BREF) (0-100%):* |  |  |  |  |  |  |  |  |  |  |  |  |  |  |
| *Physical domain* | 72.69±12.91 |  |  | 75.95±11.36 |  |  | **0.03482** | 78.05±8.55 |  |  | 76.99±11.16 |  |  | 0.4186 |
| *Psychological domain* | 63.20±13.82 |  |  | 65.28±11.14 |  |  | 0.1159 | 69.31±8.97 |  |  | 68.66±9.32 |  |  | 0.6191 |
| *Social relationships domain* | 66.87±15.50 |  |  | 69.09±14.25 |  |  | 0.1839 | 70.52±11.19 |  |  | 67.88±12.72 |  |  | 0.0683 |
| *Environment domain* | 66.55±11.67 |  |  | 68.60±10.50 |  |  | 0.0855 | 71.19±10.49 |  |  | 72.11±9.07 |  |  | 0.415 |
| *Mean* | 67.33±11.04 |  |  | 69.73±8.73 |  |  | **0.0085** | 72.27±7.89 |  |  | 71.41±8.44 |  |  | 0.3101 |
| ***Sleep quality*** *- Pittsburgh Sleep Quality Index (PSQI) [n (%)]:* |  |  |  |  |  |  |  |  |  |  |  |  |  |  |
| *poor sleep quality* |  | 13 | 67.50 |  | 13 | 67.50 | 1 |  | 10 | 24.39 |  | 9 | 21.95 | 1 |
| *good sleep quality* |  | 27 | 32.50 |  | 27 | 32.50 |  |  | 31 | 75.61 |  | 32 | 78.05 |  |
| ***Global assessment test*** |  |  |  |  |  |  |  |  |  |  |  |  |  |  |
| *General state of well-being [n (%)]:* |  |  |  |  |  |  |  |  |  |  |  |  |  |  |
| *poor* |  | 0 | 0 |  | 0 | 0 | 0.8598 |  | 0 | 0 |  | 0 | 0 | 0.9491 |
| *fair* |  | 9 | 22.50 |  | 7 | 17.50 |  |  | 2 | 4.88 |  | 3 | 7.32 |  |
| *good* |  | 13 | 32.50 |  | 15 | 37.50 |  |  | 21 | 51.22 |  | 22 | 53.66 |  |
| *very good* |  | 17 | 42.50 |  | 16 | 40.00 |  |  | 16 | 39.02 |  | 14 | 34.14 |  |
| *excellent* |  | 1 | 2.50 |  | 2 | 5.00 |  |  | 2 | 4.88 |  | 2 | 4.88 |  |
| ***Life satisfaction in the last 7 days [n (%)]:*** |  |  |  |  |  |  |  |  |  |  |  |  |  |  |
| *poor* |  | 0 | 0 |  | 0 | 0 | 0.9938 |  | 0 | 0 |  | 0 | 0 | 0.9292 |
| *fair* |  | 7 | 17.50 |  | 7 | 17.50 |  |  | 3 | 7.32 |  | 4 | 9.76 |  |
| *good* |  | 21 | 52.50 |  | 20 | 50.00 |  |  | 22 | 53.66 |  | 22 | 53.66 |  |
| *very good* |  | 8 | 20.00 |  | 9 | 22.50 |  |  | 13 | 31.71 |  | 11 | 26.83 |  |
| *excellent* |  | 4 | 10.00 |  | 4 | 10.00 |  |  | 3 | 7.32 |  | 4 | 9.76 |  |
| ***Overall well-being improved as a result of taking the supplements [n (%)]:*** |  |  |  |  | 6 | 15.00 |  |  |  |  |  | 4 | 9.76 |  |
| ***Life satisfaction improved as a result of taking the supplements [n (%)]:*** |  |  |  |  | 7 | 17.50 |  |  |  |  |  | 4 | 9.76 |  |

Bold character is displayed only for significant values (p<0.05);

^#^ Pearson's Chi-squared test is performed for categorical qualitative variables to check independence

^*^ t-test is performed for continuous variables

**Supplementary Table 5. Daily physical and sleep parameters measured by MiBand 7 watch wearable devices during the study period (from baseline (T0) to Follow up (T1)), in group G1 and group G2.**

| **MIBAND7 PARAMETERS** | | **G1** | **G2** |  |
| --- | --- | --- | --- | --- |
|  |  | **Mean ± SD** | **Mean ± SD** | **Mann-Whitney U test**  ***p*** |
| ***PHYSICAL PARAMETERS*** | ***Steps (n)*** | 8492.6±2493.1 | 8598.1±2121.6 | 0.7628 |
|  | ***Distance (m)*** | 5814.6±1842.2 | 5969.3±1660.6 | 0.6330 |
|  | ***Run distance (m)*** | 4629.3±1401.8 | 4728.4±1231.0 | 0.6408 |
|  | ***Calories (kcal)*** | 228.8±151.9 | 214.5±138.5 | 0.788 |
| ***SLEEP***  ***PARAMETERS*** | ***Total sleep time (min)*** | 442.6±39.1 | 431.1±42.1 | 0.1634 |
|  | ***Deep sleep time (min)*** | 74.7±10.6 | 73.7±10.8 | 0.5422 |
|  | ***Shallow sleep time (min)*** | 286.05±29.9 | 278.8±32.7 | 0.2502 |
|  | ***REM time (min)*** | 74.3±19.9 | 71.1±22.1 | 0.8932 |

**Supplementary Figures**

**Supplementary Fig 1. In vitro effect of *Monarda didyma* L. extract solution on DNAmAge.**

*In vitro* effect of *Monarda didyma* L. extract solution on DNAmAge in human dermal fibroblast. The quantification of DNAmAge, calculated according to the Skin and Blood Clock, is reported in years. Data are reported as mean ± SEM Student t-test was used for statistical analysis. *, p<0.05.

**Supplementary Fig. 2. In vitro effect of *Monarda didyma* L. extract solution on MCP-1.**

*In vitro* effect of *Monarda didyma* L. extract solution and dexamethasone, used as a positive control, on MCP-1 levels in primary human umbilical vein endothelial cells (HUVEC). The quantification of MCP-1 levels is reported compared to control LPS in percentage (%). Data are reported as mean ± SEM. One-way ANOVA was used for statistical analysis; ****, p<0.0001.

**Supplementary Fig. 3. Comparison of total minutes of sleep per night derived from data collected by the MiBand 7 wearable device and from** **PSQI questionnaire, measured during the study period (from baseline (T0) to Follow up (T1)), in all participants of G1 and G2 group.**

| ***Total minutes of sleep per night***  **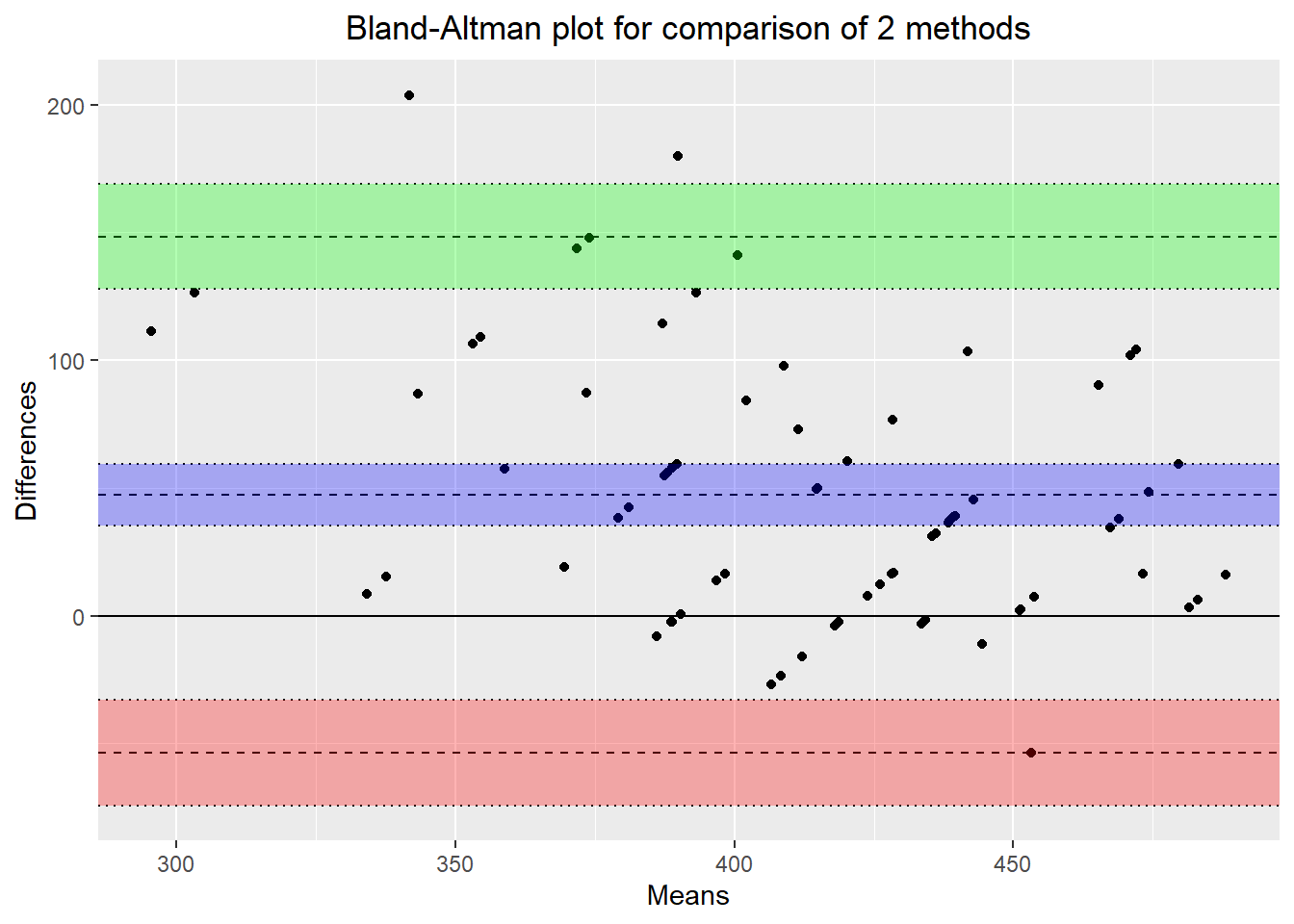**  *Differences (min)*  *Means (min)* |
| --- |

Comparison of total minutes of sleep per night obtained from the MiBand 7 wearable device and PSQI questionnaire responses is reported using Bland-Altman plot. The comparison shows variability with a mean difference of 47.44 minutes. Differences between Xiaomi MiBand7 and PSQI questionnaire data for the total minutes of sleep per night in minutes (y-axis), are plotted as a function of means, measured in minutes, of Xiaomi MiBand7 and PSQI questionnaire data for total minutes of sleep per night (x-axis). Circles represent study participants. Zero lines are marked and represent perfect agreement. Dotted lines represent bias and Bland-Altman 95% limits of agreement.

**Supplementary Fig. 4. Correlation curves between the Movement Index and the difference in biological aging indicators from follow up to baseline, i.e.** **DNAmAge T1-T0, LTL T1-T0 and Hematological age T1-T0, in G1 (A, B and C, respectively) and G2 groups (D, E and F, respectively).**

G2 group

G1 group

| **A**  p= 0.9646 | 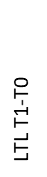**B**  p= 0.4647 | **C**  Hematological age T1-T0  p= 0.1531 |
| --- | --- | --- |
| **D**  p= 0.9410 | 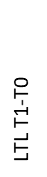**E**  p= 0.8570 | **F**  Hematological age T1-T0  p= 0.8528 |

Simple linear regression plots show the correlation in G1 and G2 groups between the Movement Index and DNAmAge T1-T0 (A, r=0.0078 and p=0.9646; D, r=0.0124 and p=0.9410), LTL T1-T0 (B, r=0.1277 and p=0.4647; E, r=-0.0302 and p=0.8570), and Hematological age T1-T0 (C, r=0,2467 and p=0.1531; F, r=0.0311 and p=0.8528). Mean, standard error (SE), and 95% coefficient intervals (CI) are represented as green, pink, and black lines, respectively.

**Supplementary Figure 5. Correlation curves between the Sleep Index and the difference in biological aging indicators from follow up to baseline, i.e. LTL T1-T0 and Hematological age T1-T0, in G1 (A and B) and G2 groups (C and D).**

G1 group

G2 group

| 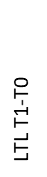**A**  p= 0.7462 | **B**  Hematological age T1-T0  p= 0.9936 | |
| --- | --- | --- |
| 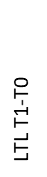**C**  p= 0.1866 | | **D**  Hematological age T1-T0  p= 0.8441 |

Simple linear regression plots show the correlation in G1 and G2 groups between the Sleep Index and LTL T1-T0 (A, r=-0.056725 and p=0.7462; C, r=0.218968 and p=0,1866), and Hematological age T1-T0 (B, r=-0.124919 and p=0.4549; D, r=-0.032997 and p=0.8441). Mean, standard error (SE), and 95% coefficient intervals (CI) are represented as green, pink, and black lines, respectively.

**Supplementary note 1**

**Randomisation of participants to intervention and control groups**

The coordinating centre and steering committee will generate the two comparison groups using simple randomization, with an equal allocation ratio, by referring to a table of random numbers. Subjects with the same age, gender, education, to avoid possible confounding variables, will be called for enrolment in groups of 10. Blocking ensures that the numbers of participants to be assigned to each of the comparison groups will be balanced within paired blocks of five in one group and five in the other for every 10 entered participants. The block size may be randomly varied to reduce the likelihood of foreknowledge of intervention assignment. The allocation sequence will be concealed from the personal staff enrolling and assessing participants in sequentially numbered, opaque, sealed and stapled envelopes. Aluminium foil inside the envelope will be used to render the envelope impermeable to intense light. To prevent subversion of the allocation sequence, the name and date of birth of the participant will be written on the envelope. Corresponding envelopes will be opened only after the enrolled participants completed all baseline assessments and it will be time to allocate the intervention.
